# Global Genome Conformational Programming during Neuronal Development Is Associated with CTCF and Nuclear FGFR1—The Genome Archipelago Model

**DOI:** 10.3390/ijms22010347

**Published:** 2020-12-31

**Authors:** Brandon Decker, Michal Liput, Hussam Abdellatif, Donald Yergeau, Yongho Bae, Josep M. Jornet, Ewa K. Stachowiak, Michal K. Stachowiak

**Affiliations:** 1 Western New York Stem Cell Culture and Analysis Center, Department of Pathology and Anatomical Sciences, University at Buffalo, The State University of New York, Buffalo, NY 14203, USA; bdecker@buffalo.edu (B.D.); liput.michal@gmail.com (M.L.); yonghoba@buffalo.edu (Y.B.); eks1@buffalo.edu (E.K.S.); 2 Mossakowski Medical Research Center, Stem Cell Bioengineering Department, Polish Academy of Sciences, 02-106 Warsaw, Poland; 3 Department of Electrical and Computer Engineering, Institute for the Wireless Internet of Things, Northeastern University, Boston, MA 02115, USA; habdella@buffalo.edu (H.A.); j.jornet@northeastern.edu (J.M.J.); 4 UB Genomics and Bioinformatics Core, University at Buffalo, The State University of New York, Buffalo, NY 14203, USA; donaldye@buffalo.edu

**Keywords:** chromatin structure, FGFR1, HoxA, embryonic stem cells, neuronal committed cells

## Abstract

During the development of mouse embryonic stem cells (ESC) to neuronal committed cells (NCC), coordinated changes in the expression of 2851 genes take place, mediated by the nuclear form of FGFR1. In this paper, widespread differences are demonstrated in the ESC and NCC inter- and intra-chromosomal interactions, chromatin looping, the formation of CTCF- and nFGFR1-linked Topologically Associating Domains (TADs) on a genome-wide scale and in exemplary HoxA-D loci. The analysis centered on HoxA cluster shows that blocking FGFR1 disrupts the loop formation. FGFR1 binding and genome locales are predictive of the genome interactions; likewise, chromatin interactions along with nFGFR1 binding are predictive of the genome function and correlate with genome regulatory attributes and gene expression. This study advances a topologically integrated genome archipelago model that undergoes structural transformations through the formation of nFGFR1-associated TADs. The makeover of the TAD islands serves to recruit distinct ontogenic programs during the development of the ESC to NCC.

## 1. Introduction

The ontogenic process begins with the pluripotent Embryonic Stem Cells (ESCs) of the blastocyst giving rise to all cell types in the body [1]. This development potential gradually becomes restricted as the cells proceed to their terminal tissue phenotypes. The ESC genome contains information to produce all types of cells, but through selective expression of cell-identity associated multi-gene programs, specific cells such as neurons are formed.

Studies in our laboratory have shown that during retinoic acid (RA) induced in vitro formation of immature postmitotic neurons (Neuronal Committed Cells, NCC) from mouse ESCs, a total of 2851 genes (20% of all expressed genes) change their activities [2]. Similarly, differentiation of human NCC from the neural stem cells alters the expression of 4704 genes [3], the majority of which bind nFGFR1 [4]. This development of NCCs is accompanied by (1) the deconstruction of coordinated gene activity networks that underwrite phenotypes of non-differentiated cells and (2) the construction of new coordinated networks underwriting cell differentiation and neuronal development. The regulated genes include the ontological categories of cell division and proliferation, development of the nervous system, development of the brain and its parts, stem cell self-renewal program, neuronal differentiation, axonal guidance and growth, synapse formation, neuronal survival, and other related categories [2,3]. Thus, our overall question is how can thousands of diverse genes at different genomic locations be coordinately expressed and regulated?

Recently, protein-mediated DNA-DNA interactions between distant chromosome regions, 100’s or 1000’s of kilobases (kb) apart and even between DNAs of different chromosomes [5,6,7] have been revealed through new Chromosome Conformation Capture (3C) and high throughput interaction assays (Hi-C and HiChIP). These interactions generate DNA loops and Topologically Associated Domains (TADs) containing compartmentalized regions of DNA that are 100’s of kb in length [6]. Within the same TAD, genes are brought into proximity and subjected to similar regulations, which are different from regulations outside the TAD borders [7].

A significant portion of DNA-DNA interactions and consequent chromatin looping events are executed by chromatin architectural protein complexes containing the CCCTC-binding factor CTCF [6]. CTCF binds convergent CTCF motifs on both sides of topological domains to mediate long-range DNA looping events between them [7]. CTCF participates in delineating boundary regions that separate transcriptionally associated genomic loci from non-related locations [7]. Studies focused on CTCF and its relationship to TADs have advanced a model in which one of the physical causes of DNA loop extrusion is the binding of CTCF [8]. Although CTCF activity has been shown to contribute substantially to TAD organization, other pathways may exists that could lead to TAD formations but these have been only sparsely studied. Transcription factors (TF) and coregulators have been proposed to contribute to the TAD formation in addition to their individual gene regulation activity [9,10]. In the present investigation we inquired about the potential relationships of chromatin looping to several ontogenic TFs with a focus on ontogenic signaling protein, the nuclear form of Fibroblast Growth Factor Receptor 1 (nFGFR1) [11,12,13,14].

Mutations of the FGFR1 gene interferes with gastrulation, as well as with the development of the neural plate, neural crest, central nervous system, and somites by affecting the expression of diverse groups of genes [1] and microRNAs [15]. These observations placed FGFR1 at the top of the developmental gene hierarchy. Studies in our laboratory revealed a new type of pan-ontogenic mechanism, Integrated Nuclear FGFR1 Signaling (INFS), which affects diverse genome ontogenic programs [1,2,3,4,16]. The highly regulated nuclear translocation of nFGFR1 integrates signals from RA and other development-controlling factors including BMP7, NGF, vitamin A, estrogen receptor [17], neurotransmitter acetylcholine, angiotensin, and cell contact signaling molecules, and targets thousands of genes encoding mRNAs, as well as noncoding miRNAs. RA induces the accumulation of nFGFR1, and is capable of directing the differentiation of ESC into cell types of different germ layers dependent on the RA concentration [18]. RA is an early embryogenesis signaling molecule which controls body-axis patterning and organ development through the Retinoic Acid Receptor (RAR) and the Retinoid X Receptor (RXR) which complex with nFGFR1 [19,20].

Nuclear accumulation of newly synthesized FGFR1 is enabled by a unique transmembrane domain and is mediated by Fibroblast Growth Factor 2 (FGF2) and β-Importin and has been shown accumulated in the nucleus of various cell types using an array of different FGFR1 antibodies, and in live cells transfected with FGFR1-EGFP [1]. nFGFR1 accumulation is increased in developing brain cells during neuronal differentiation of ESC to Neuronal Progenitor Cells (NPC) [1]. Additionally, nFGFR1 is overexpressed in diverse cancer cells contributing to their phenotypes and invasiveness [12,17,21].

Nuclear FGFR1 accumulation is both essential and sufficient in promoting neuronal development. Transfection with tyrosine kinase deleted dominant-negative FGFR1(SP-/NLS/TK-), targeted specifically to cell nucleus with the replacement of its signal peptide domain with the FGF2 nuclear localization signal, blocks neuronal differentiation of human and mouse stem cells in vivo and in vitro induced by cAMP, BMP4 or RA. On the other hand, constitutively active nuclear FGFR1(SP-/NLS) is capable of inducing neuronal differentiation of cultured stem cells, in vivo brain stem cells, and neuroblastoma cancer cells [22,23]. Both dominant-negative and constitutively active nFGFR1 affect coordinated gene activity networks of diverse ontological categories and pathways including pluripotency genes, Hox genes, diverse neurodevelopmental genes, and mesodermal genes [1,23].

Nuclear FGFR1 interacts with the common transcription coregulator CBP and binds to thousands of conserved loci of the mouse and human genome [2,4,23]. Over 85% of the genes which are regulated during the ESC to neuron differentiation, or dysregulated in the neurodevelopmental disorder schizophrenia, are targeted by nFGFR1 [2,4]. Global nFGFR1 genome targeting and its dysregulation was shown in breast cancer cells in which nFGFR1 binding to estrogen receptors mediates resistance to estrogen deprivation [17]. nFGFR1 targets predominantly promoter and enhancer regions [2,4]. While nFGFR1 binding at promoters/enhancers was shown to regulate gene transcription [1,13,23], the role of non-genic nFGFR1 remains unknown [2,4]. Among nFGFR1 targeted motifs, CTCF was identified [2,4] suggesting that nFGFR1 could play a role in chromatin organization and formation of TADs.

These current studies aimed to identify general structural features of the chromatin, their remodeling, and relation to genome functional programming during ESC to NCC development. We inquired on the roles of nFGFR1 and its partner CTCF in TADs delineation as a mechanisms for coordinated transcriptional regulation on a genome wide scale and in exemplary Hox loci. The results reveal distinct structural roles for CTCF and nFGFR1 in developmental chromatin dynamics and functional programming.

## 2. Results

### 2.1. High-Throughput Interaction Analysis Points to a Vast Complexity of Genome Organization and Remodeling during Neuronal Development

With the advancement of 3C methods [5,7] that reveal examples of interactions between distant DNA loci, the concept that the genome functionality may be explained through chromatin interactions has advanced into the forefront of scientific debate. The purpose of this study was to characterize the relationships between global genome interactions (the DNA interactome) and gene expression. The study explored the fundamental phase of neuronal development, differentiation of the ESCs to NCCs and the roles of two proteins, the pan-ontogenic genome programmer nFGFR1 and the architectural protein CTCF in chromatin structural organization and remodeling.

Towards these goals, mouse ESC and NCC genomes were processed together so that genome attributes could be compared for correlated activities. We used Hi-C and HiChIP data (Table S1 Sheet 2) generated in the current investigation, ChIP-seq, RNA-seq databases generated earlier in our laboratory [2], and DNA structural characteristic and DNA binding motif information from publicly available datasets [24,25,26].

**Figure 1:**
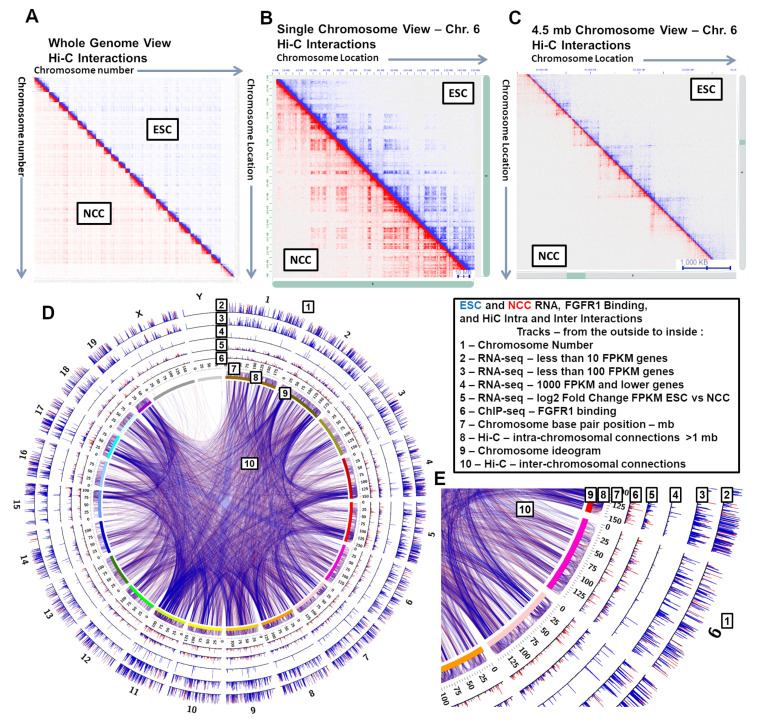
Chromatin interactions occur across the genome in both ESCs and NCCs. (**A**) Full genome inter- and intra-chromosomal contact map. (**B**) Chromosome 6 intra-chromosomal contact map. (**C**) 49.5–54.0 mb chromosome 6 contact map. (**D**) Genome-wide data overviews of RNA-seq and nFGFR1 binding and interactions in ESCs and NCCs. (**E**) Data overviews at Chromosome 6 for RNA-seq, nFGFR1 binding, and interactions in ESCs and NCCs. Values are shown for ESCs in blue and NCCs in red.

To assess the quality of the ESC and NCC interactomes, we analyzed valid interaction reads against randomized shuffled interaction anchors and a control that predicts expected interactions based on read density [27]. The Hi-C libraries showed distinct structures at whole genome, single chromosome, and 4.5 mb genomic spans (Figure 1A–C). In contrast, the random shuffled interaction locations displayed repeated patterning with no distinct structures, and the expected control interactions showed only very close range interactions (Figure S1A–C). Intra- and Inter-chromosomal interactions are seen within all the Hi-C and HiChIP ESC and NCC datasets. At the level of 4.5 mb genomic spans, visible differences were observed between ESC and NCC interactomes, including within the HoxA-D gene clusters. Those differences were addressed in our subsequent analyses.

A circular diagram shows genome-wide and chromosome 6 overviews of Hi-C data sets along with RNA levels (RNA-seq FPKM), and nFGFR1 binding (ChIP-seq) data generated in our earlier study [2]. The diagrams (Figure 1D,E) compare ESCs (blue) and NCCs (red) for the gene expression levels, nFGFR1 binding, and for intra- and inter-chromosomal DNA interactions. The DNA interactions are shown in the genome-wide overview in Figure 1D and in a chromosome 6 overview in Figure 1E. Interactions between chromosomes 6 and 11 are shown in Figure S1D. Additionally, chromosomal pairs are visualized with different spectral colors, a different color for each chromosome (Figure S2E), showing frequent connections formed between chromosome pairs in the ESC condition (comparable results seen with NCC interactions are not shown).

From the outside to the inside, the tracks on Figure 1D,E show chromosome number (1), three tracks (2–4) with RNA FPKM scores for three level FPKM ranges (low to high) that show genes that are expressed at different levels in ESC and NCC (total 2851 genes [2]), log2 fold change in RNA between ESC and NCC showing genes that are expressed at higher levels in NCC (total 1477 genes [2]) and genes that are expressed in higher levels in ESC (total 1384 genes [2]) (5), ChIP-seq identified nFGFR1 binding sites in the ESC genome (11,378 sites [2]) and the increased number of nFGFR1 binding sites in the NCC genome (46,137 sites [2]) (6), the base pair position locations in mb for each chromosome (7), Positions of Hi-C intra-chromosomal interactions greater than 1 mb in length (8), an ideogram marker (a different color for each chromosome (9), and the Hi-C inter-chromosomal interactions (10). The intra- and inter-chromosomal interactions often share the same (or nearby) anchor points. It can be seen that major intra- and inter- chromosomal interactions are similar in both ESC and NCC, while other specific interactions are only seen in ESC or NCC (Figure 1E tracks 8 and 10). The graphs illustrate a visible coincidence between the locations of active genes, their targeting by nFGFR1 and the interactions (intra-chromosomal and inter-chromosomal) anchor points, indicating that gene activities, nFGFR1 binding, and chromatin structure, are linked at the global genomic scale.

### 2.2. Inter- and Intra-Chromosomal Interactions Change between ESC and NCC. Close Range Intra-Chromosomal Interactions Are Favored in ESC and Longer Range in NCC

To investigate general chromatin structural differences between ESCs and NCCs, we compared interaction scores between the conditions in 1 mb by 1 mb interaction bins (Figure S3A,B). The intra-chromosomal comparisons show a direct relationship (diagonal line) for ESC-favored interactions between nearby locations across each chromosome, while the NCC-favored interactions are predominantly outside the diagonal for each chromosome. These opposite patterns indicate that the intra-chromosomal interactions are stronger between closer distances in ESCs and between longer range distances in NCCs. The inter-chromosomal preferences also changed between ESC and NCC throughout the genome (Figure S3A,B). Notably the inter-chromosomal anchor points predominant in one condition (ESC or NCC) are commonly shared with multiple inter-chromosomal interactions favored for that condition using the same, or nearby, anchor point (Figure S3A,B).

To investigate overall interaction changes from ESC to NCC, network comparisons were completed by applying Cytoscape Network Analyzer tool [28] on the top 5000 ranked interactions from Figure S3A. The interactions are illustrated on circular interaction diagrams (Figure S3C). T-tests show that the prominent ESC intra- and inter-chromosomal interactions are lost and new NCC interactions are gained during NCC development.

Out of each of the top 5000 interaction networks (Figure S3C) filtering was completed to keep only the 95th percentile of interaction scores. The average clustering coefficients (CC) and node degree distributions (DD) were calculated for ESC and NCC and the differences were assessed by one-way ANOVA. Highly significant differences in CC and DD were found between ESC and NCC for ESC and NCC predominant interaction networks. ESC-intra higher-CC *p* = 1.69 × 10^−20^, DD *p* = 5.05 × 10^−20^. NCC-intra higher-CC *p* = 5.97 × 10^−5^, DD *p* = 3.48 × 10^−13^. ESC-inter higher-DD *p* = 1.15 × 10^−52^. NCC-inter higher DD *p* = 2.32 × 10^−31^ (Table S1 Sheet 4). CC and DD are indicators of network interconnectedness, and together these results demonstrate that the ESC dominant intrachromosomal and interchromosomal interactions are deconstructed and new dominant interactions form across the genome during NCC differentiation.

### 2.3. Chromatin Interaction Strength Correlates with Genome Regulatory and Coding Features, with nFGFR1 Binding and with Gene Expression Levels

**Figure 2:**
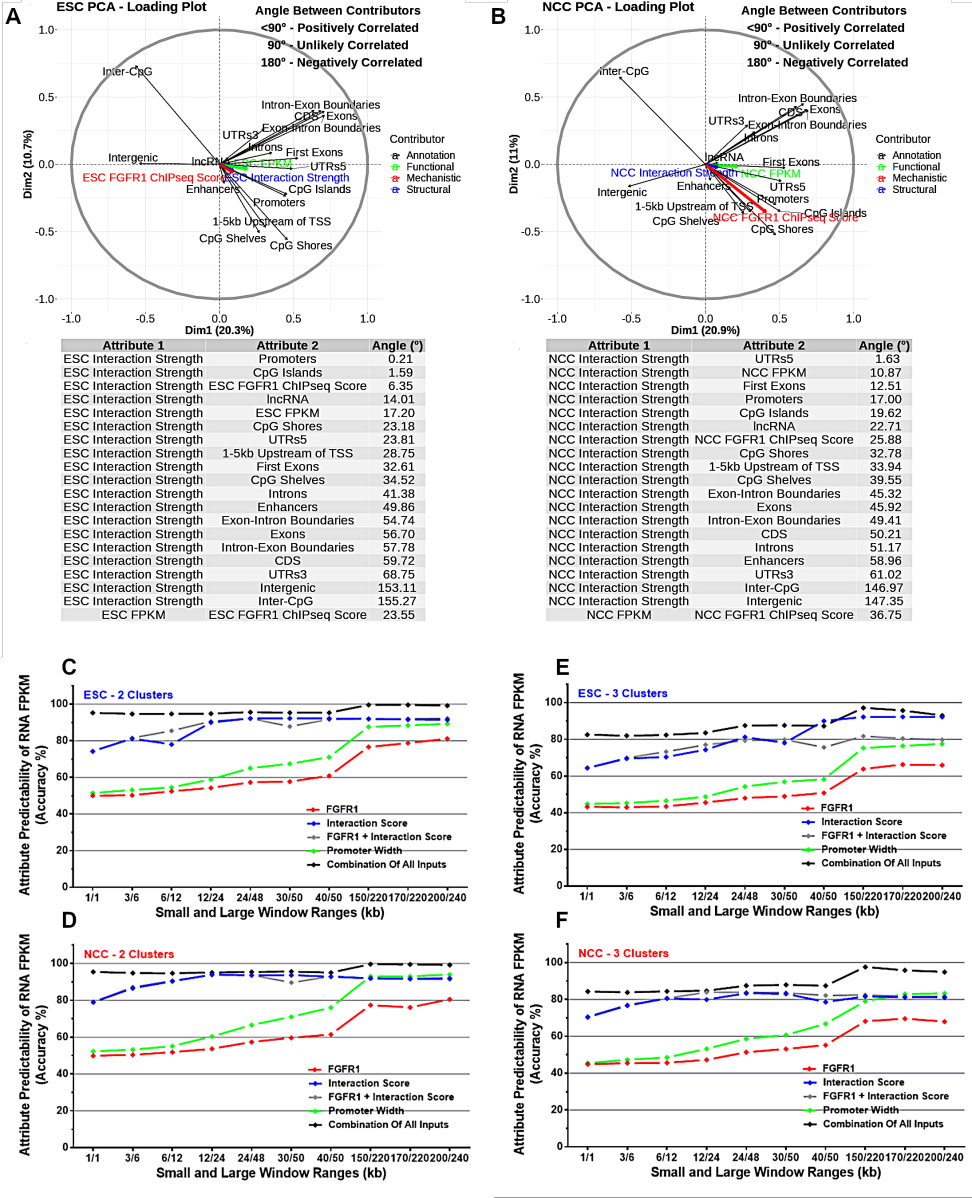
Interactions correlate with gene coding, regulatory features, and nFGFR1 binding. Interactions, gene coding and regulatory features, and nFGFR1 are predictive of RNA expression. (**A**,**B**) PCA on ESC and NCC full genome measurements of correlations between interaction anchor strength, gene coding and regulatory features, and nFGFR1 binding. (**C**–**F**) Deep Neural Network machine learning using a two-window approach prediction model of interaction strength, gene coding and regulatory features, and nFGFR1 binding for prediction of RNA expression. (**C**,**D**) Two output categories (<1 or >=1 FPKM clusters) or (**E**,**F**) 3 categories (<1, 1–30, and >30 FPKM clusters) in ESC and NCC using window-ranges of 1/1–200/240 kb were used for prediction of RNA expression from interaction strength, gene coding and regulatory features, and nFGFR1. A two-window range (ranges in kb) neural network prediction model was applied in which a smaller window as a numerator contains the attributes used for prediction (FGFR1 binding, interaction strength, genome structural features, or combinations of them), and a larger (or equal to) window as a denominator contains region within which the RNA FPKM level is predicted. Analysis was completed using Keras, to build a deep neural network capable of providing high levels of accuracy for FPKM class prediction.

To assess correlations between interaction anchor strength and genomic features on a genome-wide scale, Principle Component Analysis (PCA) was completed on ESC and NCC 1 kb binned genomes (Figure 2A,B). In both ESC and in NCC, Hi-C interaction anchor strength correlates closely with the locations of gene cis regulatory region 5’UTRs, promoters, first exons, CpG Islands, coding regions and with nFGFR1 binding strength. This indicates that the interactions of chromatin anchors are stronger and more frequent in regions containing gene regulatory features. Also, regions containing lncRNA genes are engaged in strong interactions. Conversely, chromatin interaction anchors correlated less with exons, 3’UTR and also with enhancers, although enhancers looping to promoters have been used to describe the significance of interactions [7]. Additionally, interaction anchor strength correlated negatively with intergenic regions. Importantly, interaction anchor strength correlated closely with the genome function assessed by RNA FPKM levels.

In ESC, the chromatin interaction anchors correlate strongly with the promoters and CpG Islands as well as with nFGFR1 binding locations indicating that the interaction anchors occur at or close to these locations (Figure 2A). In NCC the interaction anchors correlate more closely with the 5’UTRs and with RNA levels than in ESC, while the other high correlations (promoters, CpG islands, lncRNA sites) have less correlation than in ESC (Figure 2B). Notably nFGFR1 binding is more correlated with interaction anchor strength in ESC than in NCC, and nFGFR1 binding became less correlated to the RNA levels in NCC.

### 2.4. Machine Learning Indicates That Genome Regulatory Features and Interaction Anchor Strength Predict Gene Expression FPKM

**Figure 3:**
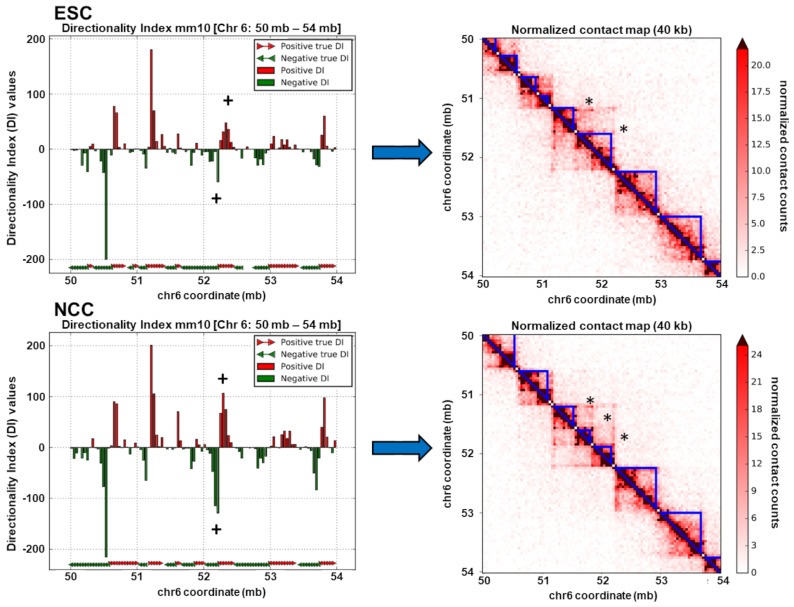
TADs occur in both ESC and NCC. ESC and NCC chr6 50–54 directionality indexes (**left**) and calculated TADs (**right**), with an example of directionality index (+) and TAD (*) reorganization during NCC differentiation.

To investigate the predictability of genome attributes in determining RNA FPKM levels, a machine learning approach was used to analyze the combined 1 kb binned genome datasets. Individual and grouped sets of attributes were used to determine different ranges of RNA FPKM using a 2-window range (ranges in kb) neural network prediction model. Two RNA output categories (<1 or >=1 FPKM clusters; Figure 2C,D) or 3 RNA output categories (<1, 1–30, and >30 FPKM clusters (Figure 2E,F) were used for prediction of RNA expression from the interaction strength, gene coding and regulatory features, and nFGFR1 binding in ESC and NCC.

The results show that combinations of several attributes (nFGFR1 binding, promoters, CpG Islands, enhancers) are highly predictive of RNA expression levels, even at smaller window ranges (1/1 kb windows give 95% predictability). The individual attributes that are not as strongly predictive in short window sizes, gain increased predictability with larger window sizes (200/240 kb windows give >75% for FGFR1 binding and >85% for promoters) (Figure 2C–F). The results indicate that these attributes not only influence control over gene expression levels in close (1 kb) range distances but also nFGFR1 can have a regulatory influence on gene expression due to its binding activities several- to hundreds of kb away from gene coding regions. When predicting interaction score we used a 150/220 kb window (Figure S4). We created two output classes based on whether the interaction score value is below or above the average interaction score. With inputs being FGFR1, Genes Promoter Width, Genes Intergenic Width, Genes 5UTRs Width, we were able to achieve a 92% accuracy on training data and 90% accuracy on testing (unseen) data. When only using FGFR1 data as input we were able to achieve an accuracy of 73% on training data, and 72% on testing data (Figure S4).

### 2.5. In ESC and NCC TADs Interaction Anchors Coincide with Gene Promoter and Coding Regions, Increase with nFGFR1 Binding, but Are Fewer in Intergenic Regions

TADs are regions of the genome which interact over the span of thousands to millions of base pairs and bring together distal genomic loci into proximal three-dimensional space [6]. TADs occur throughout chromosomes one after another, contain both + and − strand transcribed genes, and are thought to form compartmentalized functional units of the genome [6]. In the present study we calculated the locations of the ESCs and NCCs using the method of Dixon et al. [6] for 40 kb binned TAD analysis according to the protocol of Calandrelli et al. [29]. Exemplary TADs formed in ESC and NCC are shown in Figure 3 right panels.

Exemplary TADs that are remodeled in NCC are indicated by (*). Consistent with TAD remodeling, directionality indexes (+) also changed in relative strength and in some locations in direction (shown in Figure 3 left panels and also later on Figures 6E and 7E). The TADs identified in the whole genome from ESC (3965 TADs) and NCC (3953 TADs) are shown stacked on top of each other as cumulative plots in Figure S2B, with the number and sizes of TADs in ESC and NCC found to be similar, but the locations of TADs changed. Comparisons of TAD border locations between ESC and NCC showed that 38% of TAD borders changed by an average of 73,997 bp, a minimum of 40,000 bp, and a maximum of 1,600,000 bp, estimated by the distance of each ESC border to the closest NCC border. These identified TAD locations were analyzed for DNA interaction strength, genomic structural attributes, nFGFR1 binding, RNA expression levels, functional Gene Ontology categories, and DNA binding motifs.

**Figure 4:**
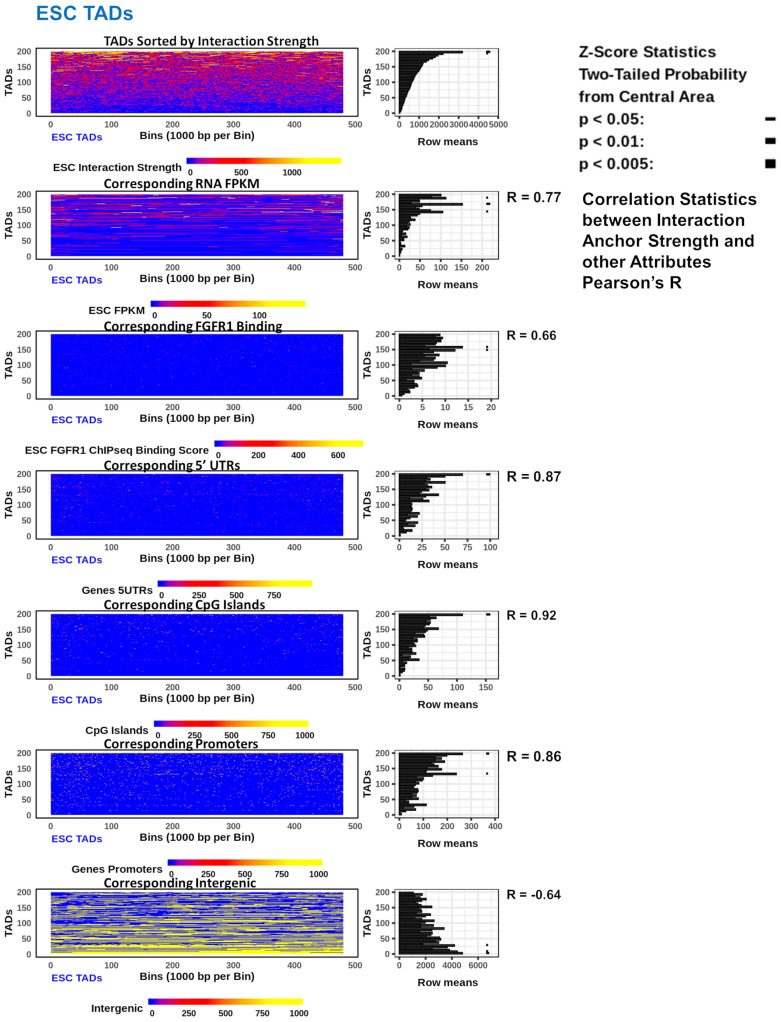
Interaction strength correlates with gene coding, regulatory features, and nFGFR1 in ESC TADs. 200 TADs of 480 kb stacked as rows in descending order by interaction strength within each TAD. Heatmaps show the feature span (gene coding and regulatory features) or strength (interaction strength, RNA FPKM, nFGFR1 binding) throughout each TAD and a mean plot of the heatmap rows in 5 TAD bins. Pearson’s R values were calculated between interaction strength and each of the corresponding attributes in 5 TAD bins. Right panels to each heatmap show average interaction strength sorted feature enrichments across same sized TADs. Z-Score statistics indicate bins which are outside the mean of all bins *p* < 0.05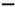; *p* < 0.01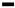; *p* < 0.005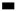.

**Figure 5:**
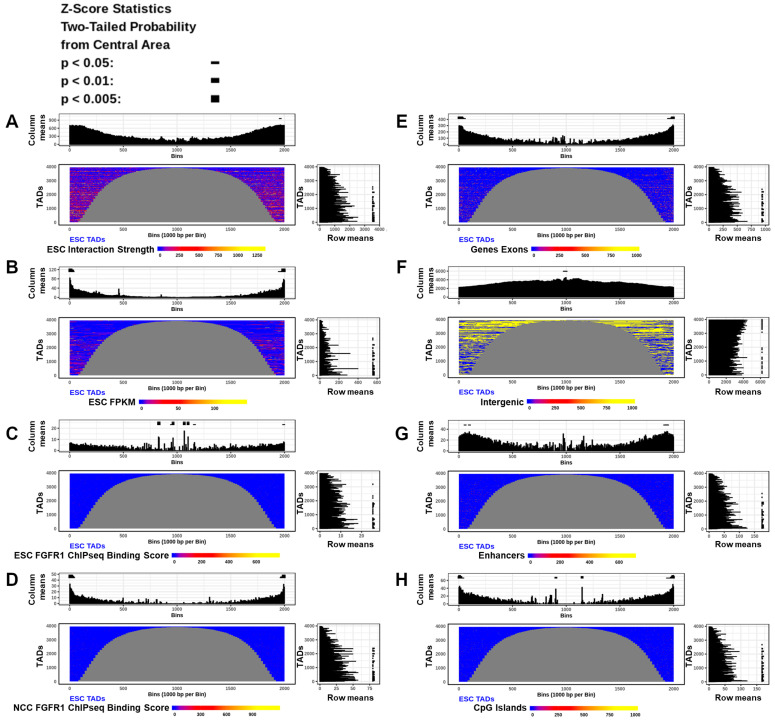
Interaction strength correlates with gene coding, regulatory features, and nFGFR1 binding in both ESC and NCC TADs. (**A**–**H**) All TADs in ESC (NCC in Figure S6) split in half and aligned by their left and right borders (NCBI directionality). Heatmaps show features enrichments in each TAD. Top and right panels to each heatmap show average location (column means—5 kb bins) and size (row means—5 TAD bins) dependent feature enrichments across all TADs. Z-Score statistics indicate bins which are outside the mean of all bins *p* < 0.05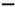; *p* < 0.01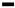; *p* < 0.005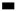.

The TADs identified in both ESCs and NCCs varied in size greatly (120 kb to 4 mb). Hence, 200 TADs of the same size, 480 kb (a common TAD size), (Figure S2C) were stacked into rows and sorted by the anchor interaction strength which was then compared to functional, mechanistic, and structural genomic features. RNA FPKM, nFGFR1 binding, promoters, CpG Islands, and 5’UTR regions were all increased with the increasing interaction strength in TADs. However, nFGFR1 binding correlates more strongly to interaction strength in NCC (R = 0.88) than in ESC (R = 0.66) (Figure 4 and Figure S5). In contrast, a negative relationship was found to exist between interaction strength in TADs and the intergenic regions, such that as the interaction strength increases, the occurrence of the intergenic regions decreases. These positive and negative correlations exist in both the ESC TADs and the NCC TADs (Figure 4 and Figure S5). Comparable results were yielded by an analysis with groupings of other sized TADs (other than 480 kb) (data not shown). Thus both genic and regulatory features of the genome sort together with interaction strength. The size of the TADs and the locations of their borders and internal regions could potentially be determined by the attributes present within. To investigate attribute enrichments in the TADs by location and size, all the identified ESC and NCC TADs were split in half and aligned by their left and right borders, with the middle regions removed for TADs greater than 2 mb (<10% of TADs). The results showed that interaction anchor strength is higher on the borders of TADs than within, and is more concentrated in smaller TADs than in larger TADs in both ESC and NCC (Figure 5A and Figure S6A). RNA FPKM is seen to be increased on the borders of TADs compared to within (Figure 5B).

The analysis of nFGFR1 binding showed that nFGFR1 binds consistently stronger in NCCs than in ESCs. In NCC nFGFR1 binds more strongly at the borders of TADs coinciding with the stronger border interaction anchor strength, however, in ESC nFGFR1 binding is less strongly bound at the borders, but more concentrated inside the TADs. (Figure 5C,D).

Investigations into the feature locations relative to TAD size showed that exons (Figure 5E), enhancers and CpG Islands (Figure 5G,H), are more concentrated in small TADs than in large TADs (Figure 5G,H). The nFGFR1 binding is stronger and the activities of the expressed genes also are higher in the small TADs. The opposite is observed with the intergenic regions which showed less concentration on the borders of TADs than within and in smaller TADs than within larger TADs (Figure 5F). The similar relations were found in ESC (Figure 5A–H) and in NCC (Figure S6A–H).

### 2.6. Interacting Genes Concentrate within TADs, Regulate Together, and Share Ontological Functions

**Figure 6:**
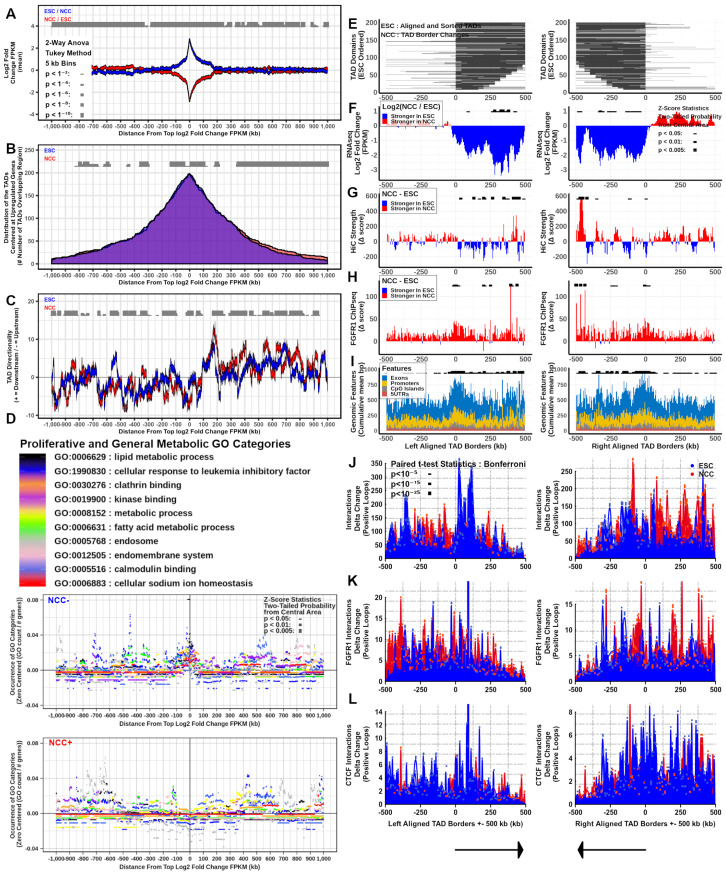
Downregulated TADs express stage specific genes and are reorganized during ESC to NCC differentiation. At interacting (q < 0.001) upregulated and downregulated anchor-anchor midpoints: (**A**) Differential gene expression. (**B**) TAD overlap. (**C**) Directionality Index. (**D**) Gene Ontology Category Enrichment. Two-way ANOVA Tukey Method: ESC versus NCC for each location shown, *p* < 1^−2^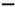; *p* < 1^−4^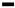; *p* < 1^−6^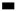; *p* < 1^−8^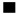; *p* < 1^−10^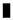. At TAD borders aligned left and right based on regulated gene directionality (adjusted *p* < 0.05): (**E**) TAD reorganization. (**F**) Differential gene expression. (**G**) Differential interaction anchor strength. (**H**) Differential nFGFR1 binding. (**I**) Differential gene coding and regulator feature enrichment. Z-Score statistics indicate bins which are outside the mean of all bins *p* < 0.05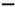; *p* < 0.01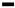; *p* < 0.005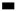. (**J**) Differential chromatin looping. (**K**) Differential nFGFR1 looping. (**L**) Differential CTCF looping. Paired *T*-Test Bonferroni adjusted *p* < 10^−5^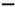; *p* < 10^−15^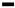; *p* < 10^−25^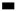.

**Figure 7:**
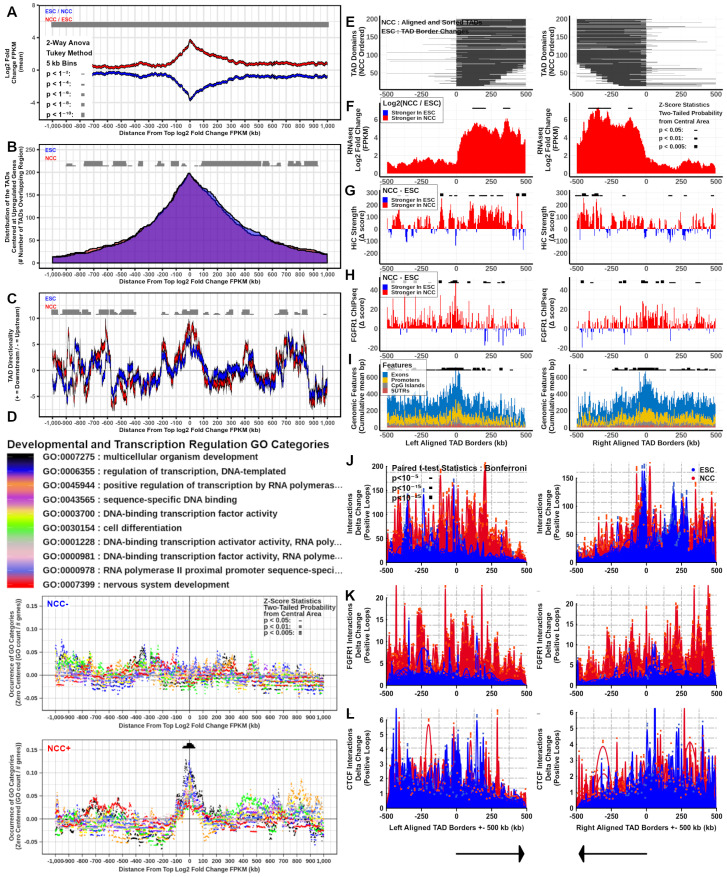
Upregulated TADs express stage specific genes and are reorganized during ESC to NCC differentiation. At interacting (q < 0.001) upregulated and downregulated anchor-anchor midpoints: (**A**) Differential gene expression. (**B**) TAD overlap. (**C**) Directionality Index. (**D**) Gene Ontology Category Enrichment. Two-way ANOVA Tukey Method: ESC versus NCC for each location shown, *p* < 1^−2^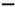; *p* < 1^−4^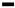; *p* < 1^−6^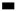; *p* < 1^−8^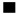; *p* < 1^−10^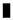. At TAD borders aligned left and right based on regulated gene directionality (adjusted *p* < 0.05): (**E**) TAD reorganization. (**F**) Differential gene expression. (**G**) Differential interaction anchor strength. (**H**) Differential nFGFR1 binding. (**I**) Differential gene coding and regulator feature enrichment. Z-Score statistics indicate bins which are outside the mean of all bins *p* < 0.05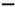; *p* < 0.01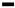; *p* < 0.005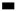. (**J**) Differential chromatin looping. (**K**) Differential nFGFR1 looping. (**L**) Differential CTCF looping. Paired *T*-Test Bonferroni adjusted *p* < 10^−5^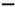; *p* < 10^−15^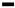; *p* < 10^−25^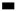.

The inherited blueprint of the genome is realized through the expression of its multi-gene programs, which during ESC to NCC differentiation involves an upregulation of 1477 and downregulation of 1384 mRNA genes and >100 (up and downregulated) noncoding RNAs [2]. To investigate the relationship between chromatin interactions and gene expression levels within TADs, we selected interacting locations containing co-regulated genes in both its anchors. For both ESCs and NCCs, interactions (q < 0.001) were ranked by the sum of log2 fold change FPKM (NCC/ESC for upregulated NCC+ genes and ESC/NCC for downregulated NCC-genes) contained within the two anchor site ranges of each interaction. The top 200 upregulated (NCC+) and top 200 downregulated (NCC−) differentially regulated interacting genes (Diffgenes) (one per 2.5 million bp) were aligned by the midpoints between their two anchor site locations (Figure S2G). These interacting regulated gene locations were analyzed together and compared with chromatin structure attributes in ESC and in NCC. Within TADs, both the top NCC+ and NCC-genes are surrounded by other genes regulated in the same direction in their nearby 5’ and 3’ (upstream and downstream by NCBI bp position notation) regions. Notably, the NCC+ co-regulated genes show larger span distances than the NCC− co-regulated genes. Also, the NCC+ co-regulated genes that are close together showed a tendency to be regulated more strongly than the NCC− co-regulated genes (Figure 6A and Figure 7A).

Investigations into the TADs which overlap the Diffgene midpoints (NCC+ and NCC− DiffTADs) showed that the majority of both the NCC+ and NCC− Diffgene midpoints are contained within TADs (Figure 6B and Figure 7B). The genome locations of the DiffGene midpoints and DiffTAD borders used in these analyses are shown in Table S1 Sheets 5–6. ESC and NCC differ in overlapping TAD start and end borders even though the average distributions are similar (Figure 6B and Figure 7B). The nearby (+/−100 kb) and distal (+/−1 mb) regions surrounding the NCC+ and NCC− Diffgene midpoints show widespread changes in interaction directionality indexes between ESC and NCC. Both the relative strength and the direction of interactions (upstream or downstream) changed throughout the regions (Figure 6C and Figure 7C).

To inquire whether such remodeling may serve to recruit distinct ontological programs, we analyzed the potential enrichment of different Gene Ontology (GO) categories +/−1 mb from the top NCC upregulated and downregulated Diffgene aligned midpoints. The results show that proliferative and general metabolic categories are overrepresented by the ESC genes that were downregulated in NCC (+/−100 kb from the aligned locations at NCC− Diffgene midpoints), but not by genes that were upregulated in NCC (Figure 6D). In contrast, developmental and transcriptional regulation GO categories are highly enriched +/−100 kb at NCC+ Diffgene midpoints but not at the regions of the NCC—Diffgenes (Figure 7D). Also, neuronal development and neuronal GO categories are significantly overrepresented within 100–200 kb at NCC+, but not at NCC− Diffgene midpoints (Figure S7C). We conclude that changes in chromatin interactions during ESC to NCC differentiation recruit genes of different ontological programs. The results advanced a “Genome Archipelago Model” (see Discussion) in which the interactions create islands (TADs) of shared functions throughout the genome which define different ontogenic programs.

### 2.7. TAD Boundaries, Interaction Strength, and nFGFR1 Binding Change as TADs Genes Are Co-Regulated during Neuronal Development

Several recent studies have found that the binding affinities of proteins involved in chromatin structure and gene regulation (i.e., CTCF, cohesin, modified histones) can be upregulated at the borders of TADs [6,7,30]. To investigate nFGFR1 binding characteristics within NCC upregulated and downregulated gene containing TADs, Diffgene midpoint overlapped TADs (DiffTADs) were right and left aligned by their TAD boundaries. The alignment was based on the average 5’ to 3’ directionality of upregulated or downregulated TAD genes. The ESC and NCC DiffTADs were sorted by their total bp lengths (smallest to largest length TADs). Calculations of differential (NCC vs ESC) TAD location span, gene expression, interaction strength, and nFGFR1 binding, we re performed in +/−500 kb ranges around both the NCC− and NCC+ DiffTAD aligned borders (Figure S2G). The results show widespread TAD reorganization during ESC to NCC differentiation illustrated by the changes in location of the TAD borders and in the interaction strength at the TAD borders and within (Figure 6E,G and Figure 7E,G). Analysis of differential gene expression shows that genes are regulated in the same direction together within the same TADs. Gene regulation is strictly defined by the borders of NCC− DiffTADs, while the borders of NCC+ DiffTADs allow for a greater spread of gene expression into the neighboring TADs (Figure 6F and Figure 7F).

Differential gene expression and interaction anchor strength together show that the changes in the interaction anchor strength and gene expression occur in parallel during ESC to NCC differentiation (Figure 6F,G and Figure 7F,G. In NCC− DiffTADs gene expression declines with the decreases in interaction anchor strength while in NCC+ DiffTADs the gene upregulation takes place alongside an upturn in the interaction anchor strength. Within DiffTADs, changes in the interaction anchor strength occur as major spikes that alternate with smaller changed or opposite direction spikes at nearby locations (Figure 6G and Figure 7G). nFGFR1 binding is markedly augmented in NCC compared to ESC in most regions of the +/−500 kb NCC− as well as NCC+ DiffTAD genomic regions surrounding the TAD borders (Figure 6H and Figure 7H). The strong increases in differential NCC nFGFR1 binding at the borders of NCC− and NCC+ DiffTADs indicates a role for nFGFR1 in border formation (Figure 6H and Figure 7H). Along with NCC nFGFR1 binding at the DiffTAD borders, the NCC nFGFR1 binding is stronger inside than outside NCC− DiffTADs. However, the opposite is observed in NCC+ TADs where NCC nFGFR1 binding outside is stronger than inside. Similar to nFGFR1 binding genomic attributes, exons, promoters, CpG islands, and 5’UTRs are strongly overrepresented on the borders of both NCC+ and NCC− DiffTADs (Figure 6I and Figure 7I). These DiffTAD analyses are also shown hichipper thresholded for HiC interaction strength by q < 0.001 and for 400 locations instead of 200 in Figure S7A,B.

### 2.8. nFGFR1 Bound Loops Are Enriched in NCC and CTCF Bound Loops Are Enriched in ESC

We used HiChIP [30] to identify nFGFR1- and CTCF- containing loops and their representation in global chromatin structures. The strength of nFGFR1- or CTCF- associated interactions were determined in all ESC and NCC TADs and separately in upregulated and downregulated gene DiffTADs. Toward this end we used split TAD analysis (Figure S6I–N) and +/−500 kb range DiffTAD aligned borders of the NCC− and NCC+ differential looping analysis (Figure 6J–L and Figure 7J–L).

Similar as with the global HiC-determined interaction anchor strength, nFGFR1 and CTCF associated interaction anchors are stronger on the borders of TADs, than within TADs (Figure S6I,J,L,M). Delta change for ESC-NCC CTCF interaction anchor strength shows that CTCF interaction anchors are more prevalent near the borders of TADs in ESC, with NCC overrepresented CTCF interaction anchor strength locations mainly occurring within the TADs (Figure S6K). Conversely, delta change for NCC-ESC nFGFR1 interaction anchor strength shows that nFGFR1 interaction anchors occur preferentially in NCC both on the borders and within TADs (Figure S6N).

Analysis by differential looping within +/−500 kb from DiffTAD borders, using delta change ESC-NCC and NCC-ESC, further reveals the remodeling of global (HiC), nFGFR1-containing (nFGFR1 HiChIP) and CTCF containing (CTCF HiChIP) loops during ESC to NCC development (Figure 6J–L and Figure 7J–L). Hi-C looping changes between ESC and NCC in both NCC− and NCC+ DiffTADs, with loops in the ESC condition replaced by new loops at adjacent locations in NCC (Figure 6J and Figure 7J). Differential looping analysis shows nFGFR1 looping to be prevalent in the NCC with the NCC enriched looping regions occurring outside and within the DiffTAD borders, with less contributions seen of nFGFR1 looping to ESC loops in NCC+ and NCC− DiffTADs, a finding that indicates that nFGFR1 is more associated with NCC chromatin looping but also maintains minor associations with ESC chromatin organization (Figure 6K and Figure 7K). Differential looping for CTCF shows CTCF looping is prevalent in the ESC with strongly ESC enriched looping areas occurring in proximity to and inside the DiffTAD borders. The CTCF looping contributes less to the NCC loops in both NCC+ and NCC− DiffTADs. Together this indicates that CTCF contributes more involvement to ESC chromatin structure while still showing some associations with NCC chromatin organization (Figure 6L and Figure 7L). Thus, during ESC to NCC transition, the loops within and outside both TADs containing up and downregulated genes are remodeled, with the replacement of CTCF-associated loops by the nFGFR1-associated loops. The analysis of FGFR1 and CTCF associated loops that form in the Hox gene clusters (Figure S16) is discussed further below.

### 2.9. CTCF, MYC, MAX, NFIC, NFKB1, Pdx1, Spz1, and ZEB1 Binding Motifs Are Overrepresented on All TAD Borders, Other TFs Motifs Are Enriched Specifically on NCC+ or NCC− DiffTADs and Several Are Targeted by nFGFR1

Several proteins are known to be associated with TAD formation including CTCF, cohesin, WAPL, and PDS5 [31]. A role for DNA binding transcription factors (TFs) in the formation of TAD boundaries has also been suggested based on their participation in the formation of the enhancer-promoter and promoter-promoter connecting loops [32]. To identify the most overrepresented TF binding motifs at and nearby the NCC upregulated and downregulated DiffTAD borders we analyzed TF binding motifs +/−195 kb from DiffTAD aligned borders in 10 kb bins using R-Gadem [24] and R-MotIV [25] (Table 1 and Figures S8–S12). Full results are presented on Figures S8–S11. Selected examples graphed individually for each DiffTAD border are shown in Figure S12. A compilation of the results for all the motif analyses together, including FGFR1 motif targeting discussed below (Figure S13), are shown in Table 1.

The results show a vast assortment of protein binding motifs overrepresented in DiffTAD borders, some of which differ between NCC− and NCC+. Multiple motifs are overrepresented at all DiffTAD borders (CTCF, MYC-MAX, NFIC, NFKB1, Pdx1, Spz1, ZEB1; Table 1 Rows 1–7) indicating their general or baseline role in TAD formations. Many more motifs were overrepresented in some, but not, all DiffTAD borders (Table 1 Rows 8–73). Several pluripotency associated TF motifs are significantly, and uniquely, overrepresented at DiffTAD borders of NCC− including (NF.kappaB, Pou5f1, CEBPA, Foxd3, Klf4, NFYA, Nkx3.2, PBX1, Sox2, T) (Table 1 Rows 94–102, and Figures S8, S9, and S12 top). In contrast, TF motifs known to control neuronal development (SOX10, TFAP2A, Nr2e3, Hand1, Tcfe2a, FOXI1, Mafb, NR3C1, Pax4, Pax5, RORA1, Regulation of transcription factors by neuronal activity, CREB1, Evi1, FEV, FOXF2, MZF15.13, NFIL3, NHLH1, Pax5, RREB1, Sox17) [33,34,35] are uniquely overrepresented at the borders of NCC+ DiffTADs indicating these TFs contribute to TAD formation in NCC (Table 1 Rows 74–93, and Figures S10–12 bottom). Together the results indicate that specific types of TFs delineate, and thus likely organize, different types of TADs through which the ESC and NCC genome programs are activated or deactivated. In addition, 20 bp sequences overrepresented in each 10 kb bin were calculated to show conserved short DNA sequences which contribute to ESC and NCC DiffTAD formations (Table S1 Sheets 7–10). These sequences were largely different in ESC and NCC and between 5’ and 3’ borders ( 99% unique) further illustrating changing TAD locations during development.

nFGFR1 which binds the common transcription coactivator CBP [23,36], has been shown to target a plethora of TF motifs, in both the mouse and the human genomes [2,37]. The TF motifs overlapped by nFGFR1 ChIPseq narrow peak binding sites were identified in 200 kb bins surrounding the NCC− and NCC+ DiffTAD Borders (+/−100 kb) using R-Gadem and R-MotIV (Figure S13). Although several of the nFGFR1 targeted motifs are shared between NCC− and NCC+, the variety of the nFGFR1 targeted motifs at the DiffTAD borders in NCC− is greater than in NCC+ (Figure S13). Several motifs were prominently identified in multiple borders (INSM1, SP1, Pax5, Klf4, Egr1), indicating their function as core sites for FGFR1 access to TADs. Importantly nFGFR1 TAD border loading into the pluripotency TF sites was observed only in ESC (except Klf4 site which is targeted by nFGFR1 in ESC and NCC). In contrast, nFGFR1 targets motifs of the NCC+ DiffTADs predominantly in NCC. These findings are consistent with the earlier findings that nFGFR1 controls (represses) pluripotency TF genes in ESC and activates neuronal genes in differentiating NCC [2]. nFGFR1 binding to these genes was verified also in human NCC [4].

In summary, FGFR1 targets several TFs motifs overrepresented in DiffTAD borders and thus could be involved in DiffTAD remodeling (Table 1 and Figure S13).

### 2.10. Chromatin Interactions, nFGFR1 Binding, and Gene Expression Change at Hox Gene Clusters during ESC to NCC Development

The findings of the present study show that intra- and inter-chromosomal interactions are an integral part of genomic organization and developmental reorganization (Figure S3A–C, Figure 5, Figure 6 and Figure 7). During development, genes of the HoxA, B, C, and D clusters, located on different chromosomes, are co-regulated and sequentially expressed reflecting the order they are positioned in on their chromosomes (e.g., HoxA1 first followed by HoxA2, HoxA3, HoxA4⋯) [1,38,39]. All of the Hox cluster’s genes are among the strongest upregulated genes during the ESC to NCC differentiation and many are targeted by nFGFR1 [2]. Hence, we inquired if the co-regulation of the individual Hox clusters is associated with remodeling of their interchromosomal and intrachromosomal interactions.

**Figure 8:**
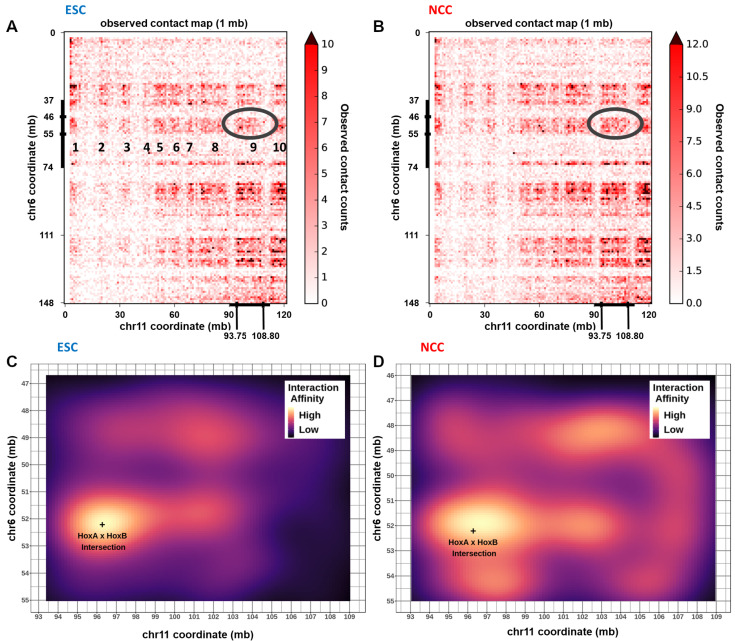
HoxA and HoxB clusters connect interchromosomally and reorganize during NCC differentiation. (**A**,**B**) Interchromosomal contact map showing locations enriched for interactions between Chr6 and Chr11 (1–10 examples numbered) with the HoxA-B interaction block circled. (**C**,**D**) Interchromosomal contact map showing enhanced view of HoxA-B interaction block with (+) indicating the intersection between the HoxA and HoxB cluster midpoints.

Our HiC analysis showed that the HoxA gene cluster on Chromosome 6 interacts with several locations (numbered 1–10) on chromosome 11 including the location of the HoxB cluster (Figure 8A,B, circled). A zoomed-in interaction map using binned interaction loop affinities (calculated using Hichipper, R-ggplot2::geom-raster), revealed enriched interactions between Chr6: 46 mb–55 mb with Chr11: 93 mb–108 mb in both ESC and NCC, which connect together the HoxA and HoxB clusters (Figure 8C,D). HoxA and HoxC (Chr15) are also connected interchromosomally in ESC and NCC (Figure S14A–D), and HoxA and HoxD (Chr 2) are connected in ESC (Figure S14E–H). The HoxA—HoxB and HoxA—HoxC interactions are maintained in NCC while the HoxA—HoxD interactions appear largely abolished. In all Hox cluster interacting blocks, the nearby interactions of the surrounding regions are remodeled (predominantly increased) in NCC. In conclusion, the HoxA cluster interacts inter-chromosomally with the HoxB, HoxC, and HoxD clusters. These interactions connect together Hox clusters during their ESC inactive state and expand to nearby regions during NCC activation.

**Figure 9:**
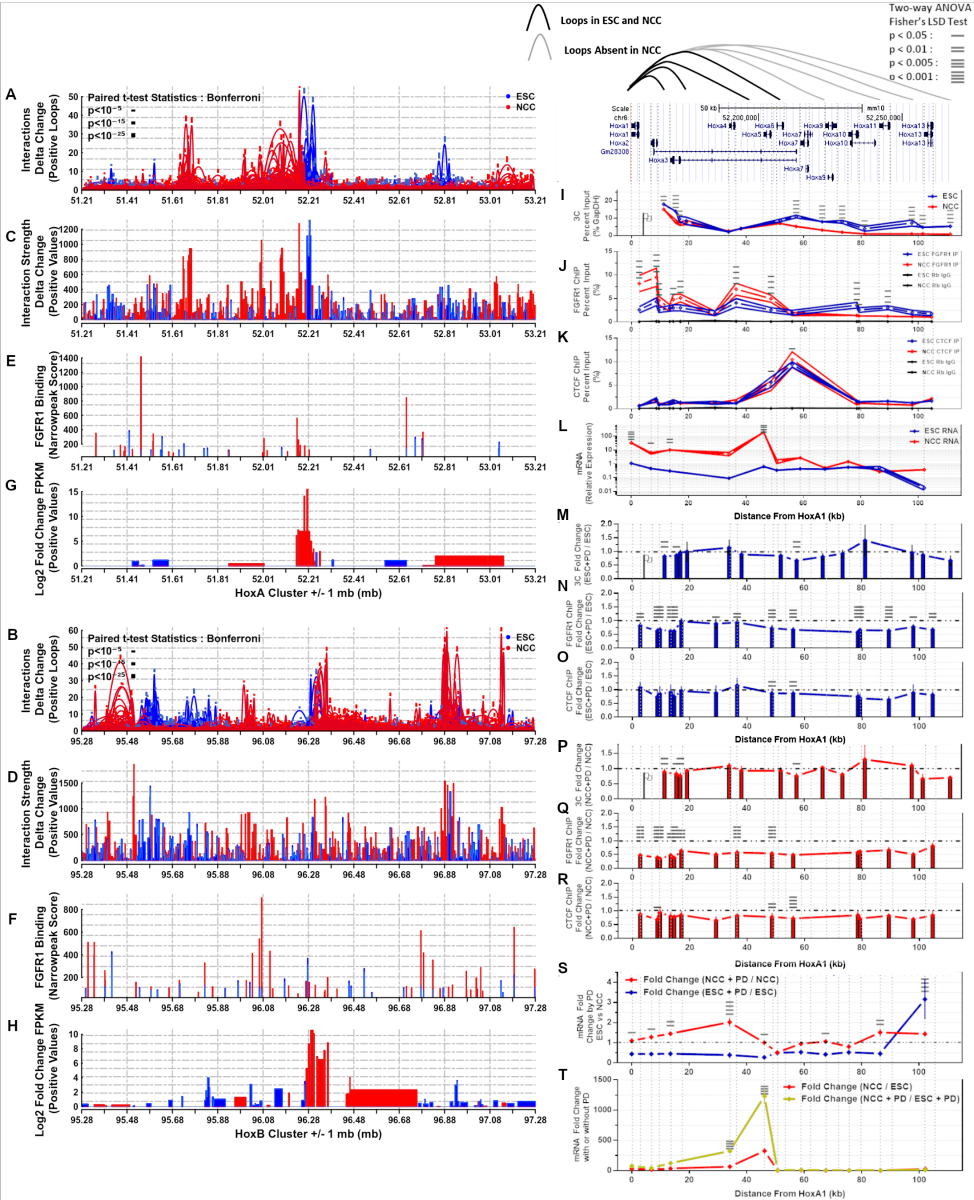
HoxA and HoxB cluster intrachromosomal interactions reorganize during ESC to NCC differentiation in parallel with gene expression and nFGFR1 binding changes. Inhibition of nFGFR1 by PD173064 in the HoxA cluster disrupts interactions at early- and mid-cluster anchor points, lowers CTCF binding, and alters gene expression. (**A**,**B**) Differential looping. Paired *T*-Test Bonferroni adjusted *p* < 10^−5^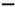; *p* < 10^−15^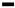; *p* < 10^−25^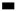. (**C**,**D**) Differential interaction anchor strength. (**E**,**F**) nFGFR1 binding. (**G**,**H**) Differential gene expression. (**I**) Interactions of HoxA1 with downstream HoxA2-A13. (**J**) nFGFR1 binding. (**K**) CTCF binding. (**L**) Gene expression. (**M**,**P**) PD fold change of HoxA1:HoxA2-A13 interactions. (**N**,**Q**) PD fold change of nFGFR1 binding. (**O**,**R**) PD fold change of CTCF binding. (**S**) PD fold change of gene expression. (**T**) ESC to NCC fold change with and without PD. Two-way ANOVA Fisher’s LSD Test: *p* < 0.05 —*p* < 0.01 =, *p* < 0.005 ≡, *p* < 0.001 ≡.

Contact map analyses of the individual HoxA/B/C/D gene clusters are shown on Figure S15A–D. Consistent with the results of the global averaged analyses described above, genes (q < 0.05 log2 fold change FPKM) that are co-regulated during ESC to NCC differentiation tend to locate in proximity to each other. In all four HoxA/B/C/D clusters, during the transition from ESC to NCC many of the the ESC Hox genes markedly increase their expression. In each Hox cluster the activation in NCC is accompanied by the loss of major nearby regions of interactions that were prominent in ESC. The ESC interactions which are absent in NCC are marked by yellow arrows (Figure S15A–D). Increased FGFR1 binding occurs in all the Hox clusters in NCC, as well as at the nearby and distal surrounding regions, in several NCC upregulated and downregulated genes. Differential looping analysis is shown alongside differential gene expression, nFGFR1 binding, and differential interaction strength within +/−1 mb from the midpoints for the HoxA and HoxB (Figure 9A–H) and HoxC and HoxD clusters (Figure S15E–L). Each Hox cluster is looped tightly together in ESC. In NCC each cluster loops out either upstream or downstream to nearby loci hundreds of kb away. The corresponding differential interaction anchor strength shows the anchor locations that are favored in either ESC or NCC (Figure 9A–D and Figure S15E–H). In NCC, new nFGFR1 binding occurs (targeting to promoters of genes) at locations as new interaction loops are formed (Figure 9E,F and Figure S15I,J). The higher gene expression locations change between ESC and NCC at and around the HoxA-D locations in parallel with the loop remodeling (Figure 9G,H and Figure S15K,L). Differential looping HiChIP analysis shows that the CTCF looping is prevalent in ESC and links sites largely within each of the Hox loci while nFGFR1 looping is prevalent in the NCC (Figure S16). Additionally, NCC enriched nFGFR1 as well as CTCF looping regions occur outside the Hox genes connecting the Hox genes to further upstream or downstream chromosomal regions. Some of the nFGFR1 and CTCF loops connect similar regions, suggesting that both proteins may co-participate in the loop formation. The significance of theses longer loops for gene regulation within the entire linked regions will be analyzed in future studies. Together the data illustrates further details of the Hox cluster reorganizations in relation to the nFGFR1 binding.

### 2.11. HoxA Cluster Quantitative Analysis: RNA Expression and Chromatin Interactions Are Delineated by FGFR1 and CTCF Binding Domains during ESC to NCC Differentiation

The experiments described above have indicated a role for nFGFR1 in the formation of the genome interactome. To further test our hypothesis, next we focused on the activation of the HoxA genes which govern the formation of different CNS regions and body parts and are activated during ESC to NCC neuronal transition [2]. The HoxA gene cluster consists of eleven HoxA genes A1, A2, A3, A4, A5, A6, A7, A9, A10, A11, A13, of which the upstream (based on NCBI bp notation) genes (HoxA1—HoxA5) are involved in the progressive (head to tail) generation of the hindbrain regions while the remaining downstream HoxA genes generate the spinal cord. nFGFR1 binds to several sites across the HoxA cluster and during RA-induced neuronal development, it activates predominantly the upstream members (HoxA1-HoxA5) of the cluster [2]. To analyze the gene interactions within the HoxA cluster we performed Chromatin Conformation Capture (3C), along with ChIP analysis of nFGFR1 and CTCF binding, and RNA expression levels (Figure 9I–L). 3C-qPCR primers were designed for the next HindIII site downstream to each nFGFR1 narrow peak ChIP-seq binding site [2] throughout the HoxA cluster. 3C-qPCRs were completed using HoxA1 as an anchor (the HoxA1 Hind III site used is marked with a flag in Figure 9I) to measure the degree of its interactions with the downstream HoxA genes. ChIP-qPCR primers were designed to each FGFR1 ChIP-seq binding site, and RT-qPCR primers were designed to each mRNA transcript of the HoxA1-13 genes. Results of preliminary 3C and FGFR1 ChIP-qPCR were reported in [37].

Two main HoxA cluster regions delineated by strong CTCF binding at the loci 48.7 and 56.0 were identified-an upstream region containing genes HoxA1–A5 (0–48.7 kb) and a downstream region containing genes HoxA6-A13 (48.7–111.1 kb) (Figure 9I–L). In ESC, HoxA1 interacted with the upstream as well as downstream region HoxA genes. In NCC the HoxA1 interactions with the upstream loci, 11.2, 15.4, and 16.8, we re partially reduced but remain unchanged for loci 19.1, 33.8, and 38.1 (Figure 9I). In contrast, HoxA1 binding to the downstream regions was reduced (loci 57.4, 66.5, 73.4) or abolished (loci 81.2, 97.7, 101.5, 111.1) in NCC. In ESC nFGFR1 ChIP-qPCR shows nFGFR1 binding to HoxA genes in both the upstream and downstream regions at similar low levels (Figure 9J). In NCC the nFGFR1 binding increased in the upstream loci 2.7, 8.7, 9.6, 14.6, 17.1, 36.5, 48.6 (Figure 9J) accompanied by upregulation of the Hox A1, A2, A3, and A5 genes (Figure 9L). In contrast in the downstream loci 78.6, 79.3, 89.3 nFGFR1 binding was reduced in NCC (Figure 9J) and expression of the HoxA6-HoxA13 genes was not significantly altered compared to ESC (Figure 9L). The CTCF ChIP-qPCR results show the strongest CTCF binding at the central 56.0 locus in ESC which was further increased in NCC while binding to the adjacent locus 48.6 became reduced (Figure 9K).

In summary, in ESC, HoxA1 engages in the interactions with all downstream, HoxA2—HoxA13, gene regions. At the midpoint of the HoxA cluster there is prominent CTCF binding (48.6–56.0) which separates differentially regulated upstream and downstream HoxA regions. During RA induced neuronal differentiation, the central CTCF binding increases, the HoxA1 locus maintains interactions only with the upstream HoxA cluster loci whereas the downstream HoxA1 loops are disassembled. Accompanying these structural changes, the upstream HoxA1–A5 region increases its binding with nFGFR1, while in the downstream region the nFGFR1 binding is reduced. As shown previously [2], during the ESC to NCC differentiation the upstream HoxA1–A5 genes become activated, while the downstream region HoxA genes maintain low activities.

### 2.12. PD173074 Inhibition of FGFR1 Reduces nFGFR1 and CTCF Binding in the HoxA Cluster Accompanied by Altered Chromatin Interactions and Gene Dysregulation

PD173074 (PD) binds in the ATP-binding pocket of the FGFR1 kinase domain [40], blocks FGFR1 autophosphorylation [41] and inhibits nFGFR1 nuclear accumulation [3,21], as well the nFGFR1 interactions with the multitude of nFGFR1 regulated genes [21]. PD shows nanomolar potency and a high selectivity for FGF receptors FGFRs [40] but is unable to block other growth factor regulated kinases or nonreceptor kinases [42]. PD173074, has the highest potency for FGFR1 (FGFR1 > FGFR2 > FGFR3 > FGFR4) [43] which also is the highest expressed FGFR gene in the ESC and NCC. PD173074’s ability to block nFGFR1 nuclear accumulation makes it an effective drug for modeling the loss of nFGFR1 activity. On the cellular level PD173074 inhibits neuronal development [3] and cell growth in a cell-type selective manner including in certain types of cancers [44].

Following 48-h of 10 nM PD exposure of LIF maintained ESC cells (Figure 9M–O), nFGFR1 binding was reduced at the loci 2.7, 8.7, 9.6, 13.4, 14.6, 17.1, 29.1, 36.5, 48.7, 56.0, 78.6, 79.3, 89.3, 97.9, 104.6 (Figure 9N), accompanied by reduced HoxA1 interactions at loci 11.2, 15.4, 57.4 (Figure 9M). CTCF binding was reduced at its central loci 48.7, 56.0 (Figure 9O).

Following 48-h of co-treatment with RA and 10 nM PD (NCC+PD) (Figure 9P–R), nFGFR1 binding reached an overall statistically significant reduction at the upstream loci 2.7, 8.7, 9.6, 13.4, 14.6, 17.1, 36.5, 48.7 (Figure 9Q) and CTCF binding was reduced at 8.7, and at the central 48.7, 56.0 loci (Figure 9R) compared to NCC. Similar as in PD treated ESC, HoxA1 interactions were reduced at upstream loci 11.2, 15.4, 16.8, 57.4 but not significantly changed at the downstream loci (Figure 9P). Thus in both ESC and NCC nFGFR1 supports the HoxA1 interactions with the upstream HoxA genes.

In ESC PD diminished nFGFR1 HoxA binding and proximal HoxA1 interactions were accompanied by markedlly reduced basal expression of the HoxA1-A11 genes. In the NCC the PD reduced FGFR1 binding and proximal HoxA1 interactions were associated with augmented upregulations of the early HoxA mRNAs (Figure 9S). These opposite changes in early HoxA mRNAs induced by PD are consistent with the nFGFR1 ability to activate as well as inhibit gene expression [2,4]. Comparing ESC to NCC differentiation with and without PD revealed a larger change in gene expression during differentiation in the early Hox genes in the presence of PD (Figure 9T). This suggests that nFGFR1 maintains gene expression at the appropriate levels at the mid and early HoxA genes during NCC differentiation.

### 2.13. nFGFR1 and CTCF Occupy Colocalized as Well as Adjacent Loci in 3D Nuclear Chromatin Space

**Figure 10:**
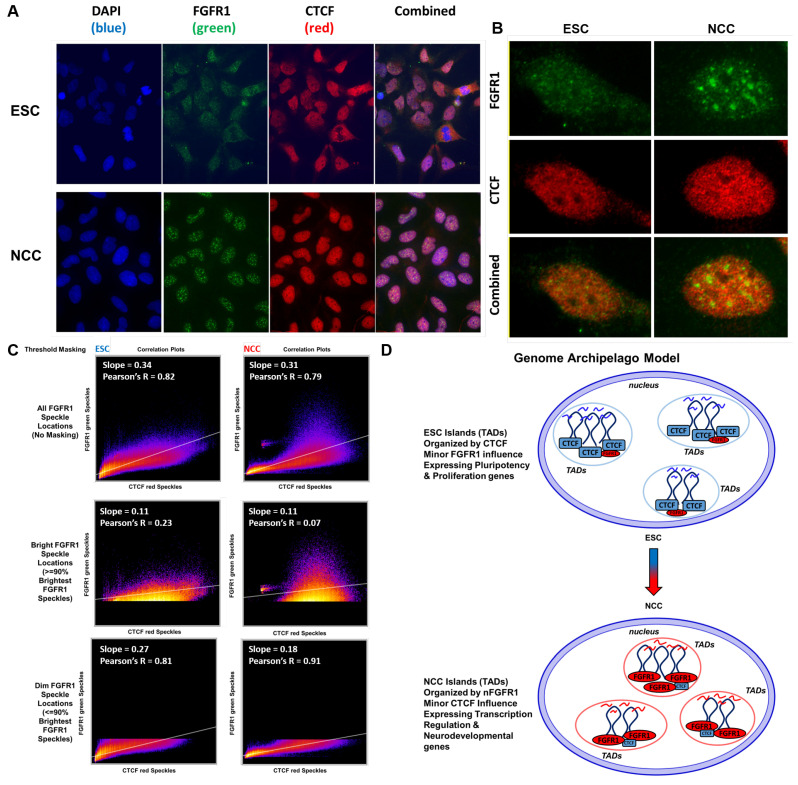
ESC nFGFR1 binding colocalizes with CTCF, NCC nFGFR1 binding targets new locations distinct from CTCF. (**A**) Immunocytochemistry of ESC and NCC stained with CTCF ab (red) and nFGFR1 ab (green). (**B**) Enhanced view of single ESC and NCC nuclei. (**C**) Correlation analysis of CTCF and nFGFR1 colocalization using Fiji ImageJ Coloc 2. (**D**) Genome Archipelago Model-hubs of transcriptional activity occur throughout the genome as TAD and multi-TAD islands. TADs containing intra-TAD genes looped together form islands alone or with other TADs which are in the vicinity of each other throughout the nucleus. TAD islands integrate multiple co-regulated genes under control of NCC− and NCC+ specific proteins, dependent on the protein binding motifs which are present. The TAD islands and looping components change during ESC to NCC differentiation. Gene co-expression relationships are conserved evolutionarily even as genes move around the genome because maintaining the same protein binding motifs allows the genes to form islands with diverse members of the same co-regulated gene group despite their distinct genomic locations. TAD island formations are controlled by CTCF and pluripotency transcription factors in ESC, and by FGFR1 and neuronal transcription factors in NCC.

Confocal images of nFGFR1 and CTCF co-immunostaining in ESC and NCC are shown in Figure 10A. An example of a single nucleus is shown in Figure 10B. In ESC nFGFR1 immunoreactivity is present throughout the cells and in NCC becomes concentrated in distinct nuclear foci shown previously to represent RNA Pol II transcription and co-transcriptional processing sites [23] (Figure 10A,B). In both the ESC and NCC nuclei one observes close localization of nFGFR1 and CTCF, yet only few prominent overlapping yellow locales form (Figure 10A,B), (Figure 10C). Despite this infrequent colocalization (18% of CTCF stain colocalized with nFGFR1 in ESC and 9% in NCC), correlation analysis (Coloc 2 in Image J Fiji) shows that the distributions of the nFGFR1 and CTCF locations are highly correlated in ESC (Pearson’s R = 0.82) and this correlation is maintained in NCC (Pearson’s R = 0.79). However in NCC, the CTCF immunostaining is largely absent from high intensity nFGFR1 foci (Figure 10C) and little or no correlation is found between the brightest nFGFR1 signals (>90% intensity) and CTCF (Figure 10C). These observations are consistent with the results of TAD analyses by showing that CTCF and FGFR1 operate at largely separate yet close chromatin locales, and that in NCC nFGFR1 accumulates in distinct chromatin domains.

## 3. Discussion

In summary, this study shows a widespread formation of intra- and inter-chromosomal loops both of which are dynamically remodeled during neuronal differentiation, along with the coordinated changes in expression of functionally related genes. Genomic interactions correlate with the gene regulatory regions and with nFGFR1 binding. Machine learning shows that chromatin interactions, nFGFR1 binding and the promoter size are predictive of gene expression levels and that their influence extends to several and to hundreds of kb away from gene coding regions. In ESC and NCC, genes are organized into TADs, due to widespread chromatin looping by genic and regulatory genome parts, and by CTCF and nFGFR1. CTCF-associated and nFGFR1-associated TAD DNA loops provide distinct structural codes for ESC and NCC genomes, respectively. The TADs interacting genes are coregulated and share ontological functions, distinct for the NCC upregulated and downregulated gene containing TADs. The NCC upregulated and downregulated gene TADs are organized by a wide assortment of pluripotency, development, and neuronal differentiation controlling transcription factors, many of which have DNA motifs targeted by nFGFR1 and some are specific for the upregulated or downregulated gene TADs. Our global genomic findings are further substantiated by the analysis of Hox genes.

The HoxA, B, C, and D genomic loci engage in widespread intra- and inter-chromosomal interactions which are reorganized during ESC to NCC differentiation along with changes in nFGFR1 binding and gene expression. The exemplary interactions within the HoxA cluster genes are delineated by CTCF binding and are controlled by nFGFR1. The outcome of our investigation is an integrated nFGFR1-engaging topological model of the genome that undergoes extensive structural remodeling as it goes through global functional reprogramming during the development of pluripotent ESC to neuronal NCC.

### 3.1. Structure and Reorganization of Genome TADs Are Associated with nFGFR1 Binding

Our current results support previous studies which have found differentiation to be accompanied by global chromatin reorganization, and that a subset of TADs are reorganized during development [9,45,46,47]. Our results show the mouse genome is composed of compartmentalized TADs, chromatin units, which occur one after another throughout each chromosome of the genome. The number of TADs is consistent with the reported ranges in mouse, human, and drosophila genomes. Dixon et al. reported 2200 TADs with a median size of 880 kb size in the J1 mouse embryonic stem cell line. Dixon also reported a 10× higher read depth than our current study and used a different mouse cell line which could account for the differences between our two studies [6]. Other studies have found > 2000 TADs or > 4000 TADs throughout the genome in different cell models [48,49]. Our work, which focused on the ESC to NCC differentiation, shows that, in these two cell types, there are similar numbers of TADs (both 4000), the TADs have similar average sizes (both averaging 625 kb), but there are widespread differences to the start and end locations of the ESC and NCC formed TADs ( 37% of TAD borders change during ESC to NCC differentiation).

Our current study investigates questions using averaged together locations to determine global characteristics of the genome. Although various useful visualization tools are already available [50,51], and similar pile-up methods have been shown previously [52], our custom analyses were designed to quantify the data specifically based on our questions of interest. Our same sized aligned TAD, and full genome aligned TAD analyses show that DNA interaction strength correlates strongly with genic (5’ UTRs, Exons) and regulatory (promoters, enhancers, CpG Islands) genome regions with gene activities and with FGFR1 binding. The strong correlations were further verified by PCA. Furthermore, Machine learning analysis showed that these regulatory and gene coding features and chromatin interactions are predictive of gene expression levels.

The study supports previous work on the general geometry of TADs whereby the frequency and strength of the TADs internal looping interactions are higher at the TAD border regions than within and that active gene expression is predominant on the borders of TADs [53]. Genic and regulatory features (Promoters, 5’UTRs, Exons, CpG Islands, and Enhancers) and FGFR1 binding (in the NCC condition) were found to concentrate close to the borders of TADs and are more concentrated on the shorter than on the longer TADs. In contrast, intergenic regions of the genome commonly make up the insides of TADs. These findings are consistent with the recent reports that in the Drosophila genome many of the TAD boundaries are present in gene-dense, chromatin-accessible, transcribed regions enriched in active chromatin marks [54], most of them occurring at active gene promoters [48]. The aligning of DiffGene containing TADs (DiffTADs) by their 5’ and 3’ borders (determined by 5’ to 3’ gene directionality) revealed that differential gene expression, differential interaction anchor strength locations, differential FGFR1 binding, and genic feature concentrations are all strongly delineated by the TAD borders. Within DiffTADs, the changes in gene expression are accompanied by sharp peaks of increased interaction anchor strength. These peaks often occur adjacent to the regions of low interaction anchor strength (or increased interaction anchor strength for the opposite ESC or NCC condition), indicating that interaction anchor points define the formation of the internal TAD loops during ESC to NCC differentiation. Such side by side adjacent interactions may represent short distance enhancer-promoter, enhancer/promoter-gene interactions described in previous literature.

By identifying highly upregulated (or downregulated) gene regions which interact with each other (NCC+ and NCC− DiffGenes) we show on a multi-locus scale that up- and down-regulated genes often occur within TADs alongside genes regulated in the same direction at nearby locations to each other. We show that TADS concentrate co-regulated genes with common biological functions. Formations of TADs has been hypothesized to serve an ontological purpose of selecting distinct gene programs for cell development, hemostasis, and other functions. The striking differences between the specific ontological categories overrepresented by NCC downregulated gene TADs (metabolism, proliferation) and by NCC upregulated gene TADs (transcription regulation, development, neuronal development) provide a strong support for this fundamental hypothesis. The development of early neurons, referred to as NCC, from the pluripotent ESC involves the formation of TADs that group genes underwriting neuron development and related functions, and deconstruction of TADs that group genes supporting active ESC proliferation. This integrated model is mechanistically supported by the tight relationship of gene expression to chromatin structure described in this section and earlier results [9,45].

Peric-Hupke et al., [55] has shown that upon differentiation of mouse ESC to neural precursor cells and astrocytes, genes involved in pluripotency are attached to the nuclear lamina and inactivated, whereas genes triggering neuronal differentiation are detached from the lamina [55]. Similar changes in gene activities have been observed in our studies [2] and are presently mapped to distinct groups of TADs: NCC− and NCC+ DiffTADs. This raised a possibility that the NCC+ DiffTADs contain genes which during RA-induced differentiation detach from the lamina and increase in gene expression.

Using the list of genes, which during ESC differentiation reduced their lamina association [55], we found that 82% of the genes were upregulated in NCC in our study. Likewise, 74% of genes which increased the lamina association [55] were downregulated in NCC. Importantly, the upregulated genes which detached from the lamina, [55], occurred 2.07-fold more often in NCC+ than in NCC− TADs. In contrast, the downregulated genes which bound to lamina were found 3.88-fold more frequently in NCC− than in NCC+ TADs. These observations warrant further investigations to ascertain if the NCC regulated TADs may form in association with the nuclear lamina.

During NCC differentiation the accumulating nFGFR1 has been shown to activate or inhibit great numbers of genes in a coordinated manner [2,37]. Here we show that as the ESC differentiate to NCC, nFGFR1 binding becomes more concentrated on NCC+ DiffTAD borders and outside TADs than within indicating that the NCC+ genes are regulated by nFGFR1 at the TAD boundaries in addition to the features described in our earlier study for nFGFR1 direct promoter regulation [36]. In NCC− DiffTADs, FGFR1 binding is concentrated also at the TAD borders, however, unlike in NCC+ DiffTADs, the FGFR1 binding concentrates at several locations inside but not outside the the NCC− DiffTAD borders. With its outside- at- and inside- border binding dynamics, nFGFR1 may potentially disrupt interactions of NCC− DiffTADs to pull them apart to make new NCC+ DiffTADs. Such TAD-based mechanisms could underwrite the synchronized global gene regulation by nFGFR1 [3] and complement the direct gene regulation by nFGFR1 binding at the proximal gene promoters shown previously [2].

Through HiChIP, we have identified the CTCF looping being associated mainly with ESC loops of the differentially regulated TADs. CTCF ESC loops primarily concentrate at DiffTAD borders (both at + and − DiffTADs), and as indicated in [7], may delineate distinct TAD boundaries and TADs separation.

In contrast, nFGFR1 associated NCC loops connect regions at, within, but also outside TAD borders, the latter indicating nFGFR1 associated loops connect the nearby TADs together. This is observed predominantly in NCC+ DiffTADs. In NCC+ DiffTADs, both the upregulated gene expression and interaction strength spread outside the TAD border edges into the next neighboring TADs, while in NCC− DiffTADs the ESC gene expression and interaction strength are sharply delineated at the TAD borders. NCC nFGFR1 narrow peak binding occurs (aside from being present on the borders of TADs) stronger on the inside of NCC− DiffTADs and stronger on the outside of NCC+ DiffTADs. These data advance a model in which nFGFR1 coordinates global gene activity by forming the TAD borders, separating TADs containing inhibited genes, looping together TADs containing activated genes, and engaging in the formation of micro-interactions (small loops). Through these actions nFGFR1 may conduct its function as a global gene regulator-coordinator.

### 3.2. nFGFR1 May Construct TADs by Targeting TAD Border Enriched TF Motifs

Consistent with the concentration of promoters and enhancers, we find concentrations of TF motifs at the TAD borders. Binding of TFs has been suggested to play a role in TAD formation, in a manner that at least in some cases appears independent of transcription as TAD formations were shown not blocked by transcription inhibitors [9,46,47]. In support of this concept, analysis of the aligned TAD borders shows that TAD 5’-3’ directionality is marked not only by border asymmetry for interaction anchor strength/looping characteristics, but also for the overrepresentation of TF protein binding motifs at the 5’ and 3’ borders on aligned TADs. Several motifs (CTCF, MYC-MAX, NFIC, NFKB1, Pdx1, Spz1, ZEB1) are overrepresented in both 5’ and 3’ TAD borders and in both NCC- DiffTADs and NCC+ DiffTADs. These motifs may organize TADs in general, by acting as organizers of a default (ground level) chromatin structure in the absence of other stage-specific developmental TAD organizers. Other TF motifs may have more specific roles in chromatin organization as they are overrepresented only in NCC-TADs or NCC+ TADs and in 5’or 3’ TAD borders. The motifs that are overrepresented only at specific left or right, NCC− or NCC+ DiffTAD borders may be important in cell type specific TAD directional organization. The proteins targeting these motifs may allow for the TADs to be regulated differently in ESC or NCC in NCC− and NCC+ DiffTADs.

One such group are TFs that form transcriptional pluripotency networks. In general, these TFs act by supporting activities of genes that drive ESC self-renewal while repressing genes that would cause ESC differentiation [56]. They are expressed in ESC and turned off in NCC [2]. Notably, we find the pluripotency TFs motifs specifically overrepresented at the 5’ borders of the NCC− DiffTADs containing the genes highly expressed in ESC and downregulated in NCC. These findings designate a new genomic mechanism for the regulation of the pluripotent state in which concentrated binding of the pluripotency TFs delineate borders of TADs that group genes involved in self-renewal, proliferation, and general metabolic functions. A mirror mechanism involving NCC+ enriched motifs may control neuronal differentiation. TFs known to promote neuronal development have their motifs uniquely overrepresented at the borders of NCC+ DiffTADs which group together the NCC upregulated genes involved in transcriptional regulation and neuronal development. Thus, TFs that control distinct ontogenic processes, may do so through a two-pronged genomic mechanism: (1) by delineating the borders of “ontogenic” TADs and (2) by their “ traditional” direct regulation of the individual developmental genes. Such dual mechanism may underwrite the coordination as well as fine tuning of the gene activities during development. We think about the formation of DNA loops and TADs as an isolation as well as grouping sections within the same DNA polymer (intra-chromosomal interactions) and between different DNA polymers (inter-chromosomal interactions).

Many of these TFs with their motifs enriched at the TAD borders are known to bind the transcriptional co-regulator CBP. CBP acts as a bridging factor that brings distant promoter or enhancer regions to the TSS [1]. By DNA binding simultaneously to different TFs and to the mediator complex CBP, CBP could bridge together the promoters of distant genes. nFGFR1 which binds CBP and promotes CBP accumulation at DNA sites [23] could control DNA looping in such a manner. In ESC and NCC nFGFR1 targets many of the same TAD border overrepresented motifs, with a higher variety of motifs targeted in ESCs, possibly indicating a general function of maintaining chromatin structures when the concentration of nFGFR1 is low in the ESCs.

The high border concentrations of genic and regulatory features and FGFR1 binding are major features of the regulated TAD formations. In the NCCs, there is a strong influx of FGFR1 into the nucleus, and increased nFGFR1 genome binding at the promoters of NCC regulated genes (FGFR1 participates in both activation and inactivation of genes) [2] throughout the genome. Our analyses show specific NCC motif binding sites where nFGFR1 binds (Arnt-Ahr, CEBPA, CTCF, E2F1, Egr1, INSM1, Klf4, Myc, MZF11.4, NFATC2, NHLH1, Pax4, Pax5, Pax6, PPARG-RXRA, SP1, SPIB, Tal1-Gata1, TEAD1, TFAP2A, TP53, Zfx, ZNF354C). Out of the motifs targeted by nFGFR1, the majority are overrepresented in NCC− and NCC+ DiffTAD borders, further supporting the role of nFGFR1 in functional control over TAD border processes. In addition, nFGFR1 predominant targeting of pluripotency TF motifs at the 5’ borders of NCC− DiffTADs and neurodevelopmental TF motifs at 5’ NCC+ DiffTAD borders, indicates that nFGFR1 may have a specific function the in delineation of TADs whose genes are repressed in NCC and activated in the NCC, respectively. This mechanism may underlie the nFGFR1 mediated ESC exit from the pluripotent state into neuronal differentiation and nFGFR1 coordinate inhibition of pluripotency and activation of neurodevelopmental gene programs. By controlling the TF genes and targeting their motifs, nFGFR1 may fine tune the NCC DiffTAD formations and functions. Together the results imply that the TAD formations occur through the combined actions of multiple proteins for the shared functions of promoter activity regulation, chromatin organization, and gene expression regulation, and that nFGFR1 is a major component in the process.

### 3.3. Hox Clusters Exemplify Global Gene Inter and Intra-Chromosomal Interactions and Their NCC Development Remodeling

Our HiC analyses show that the HoxA cluster interacts with HoxB, C, and D indicating that the clusters are in close proximity in 3D space. The results show that the clusters interact directly together in ESC and are deconstructed and expanded out to nearby regions in NCC.

Furthermore, our HiC analyses show that all Hox clusters are in intrachromosomal interaction complexes unique to ESC, which are deconstructed and replaced by adjacent interaction complexes in NCC. In the earlier study by Chambeyron and Bickmore [57], HoxB activation was shown to be associated with an increased distance between HoxB1 and HoxB9 genes, measured in 3D nuclear images. Their looping out occurs from the chromosome territory towards the center of the nucleus. The results of our HiC and 3C analyses indicate similar changes in all Hox (A,B,C,D) clusters and identify the specific interactions remodeled during ESC differentiation. These changing interactions occur in concert with increases in FGFR1 promoter binding and gene expression increases in the NCC upregulated Hox genes. DiffTAD analysis of differential looping reveals an analogous phenomenon taking place on a multi-locus scale, with regions of ESC and NCC differential looping occurring adjacent to each other, with nFGFR1 binding enriched at genic and regulatory feature enriched TAD borders, and outside (NCC+), and within (NCC-) border locations. Thus, deconstruction of the ESC loops and construction of new NCC loops in both NCC− and NCC+ DiffTADs may be executed through changes in FGFR1 binding. Based on our analyses of the Hox genes we propose a model in which, (1) in ESC, genes of the Hox clusters are looped together contributing to their low ESC activity. During NCC differentiation, FGFR1 binds to chromatin, detaching the ESC loops while promoting new interactions through its binding at the promoters of Hox genes which then become upregulated. Future studies will aim to further delineate the mechanisms by which nFGFR1 may affect the global chromatin organization.

Our experiments focused on the HoxA cluster further link nFGFR1 binding to chromatin looping. The HoxA locus is subdivided by high CTCF binding nearby to the HoxA5 and HoxA6/HoxA7 loci, into the proximal region containing HoxA1–A5 genes and the distal region containing HoxA6-HoxA13 genes. In ESC, the HoxA1 gene is connected to the downstream HoxA genes by shorter and longer loops that may include different numbers of the proximal and distal HoxA genes. In NCC, shorter loops persist, but are reorganized, while the longer loops are deconstructed. Detection of the multiple loops by 3C implies that alternating loops are dynamically formed in the ESC (short and long loops) and in NCC (short loops). Such a model is supported by single cell HiC that showed formations of alternating loops in the same cell types [58].

By reducing FGFR1 accumulation in the nucleus and its HoxA gene targeting with PD137034 (a finding consistent with PD137034 blocking of nFGFR1 nuclear accumulation and interactions with diverse genes [21]) we show that nFGFR1 supports chromatin looping at the proximal and mid loci of the HoxA cluster in both ESC and NCC conditions. The roles of loops appear however different in ESC (loops formation supports basal Hox gene activities) than in NCC (loops moderate the upregulation of HoxA genes and prevent excessive gene upregulation).

In addition, nFGFR1 depletion reduces CTCF binding consistent with the earlier shown regulation/stimulation of CTCF gene expression by nFGFR1 [2]. Thus, nFGFR1 may regulate chromatin structure by two mechanisms, by binding to HoxA genes promoters and also via CTCF by controlling its expression and binding at CTCF targeted DNA sites. nFGFR1 stabilization of the short loops, loss of FGFR1 binding at the distal HoxA7-A13 genes, and the increased CTCF insulator binding at the dividing HoxA6/HoxA7 region could all potentially act to deconstruct the longer loops in NCC. These HoxA data, offers a model in which the gain of nFGFR1 binding in NCC may stabilize the NCC loops in HoxA1–A5 and thereby prevents excessive gene activation. In ESC nFGFR1 supports both short and long loops to maintain low basal levels of gene expression.

The role of nFGFR1 in structural remodeling is shown by the PD137034-induced loss of FGFR1 function leading to the reformatting of DNA contacts. The plausible mechanisms by which nFGFR1 affects the DNA loop formation are (1) directly by delineating theTAD borders as well as (2) indirectly via regulation of genes that control TAD formation, including the CTCF gene. The nFGFR1 binds to the mouse as well as human CTCF gene promoter [4] and inhibits the CTCF gene expression [2] In addition, we showed that nFGFR1 targets the mouse and the human genomes consensus sequence that binds CTCF, and thereby may antagonize the CTCF function [2,4]. Further studies are needed to assess relative contributions of the direct and indirect nFGFR1 actions.

### 3.4. Genome Archipelago Model

As an outcome of our investigations we propose an integrated ”Genome Archipelago Model” in which TADs create transient topological islands of shared ontological functions that underwrite differentiation of ESC to NCC (Figure 10D). The changes in the TAD island formations involve increased nFGFR1 genome targeting and the replacement of the CTCF-associated ESC loops with the nFGFR1-associated loops in NCC, in which nFGFR1 remodels chromatin structure together with other architectural proteins, while lessening the influences from CTCF. This remodeling serves to recruit genes of different ontological programs, i.e., cell proliferation supported by active metabolism genes prominent in ESC gives way to transcriptional regulation of the neurodevelopmental genes in NCC. In our current study of global conformational programming we extend on the concept of transcription factories [59], to involving TAD and multi-TAD domains. We refer to these functionally related structural domains, as archipelagos of islands. We further propose a genome wide organization of ontogenic programs based on the TADs islands and their dynamic remodeling during cell development.

Several analyses in this study contribute data to the model. Figure 6D and Figure 7D show that genes of shared ontogenic functions that are inside TADs are regulated together across the genome during development. Figure 6E–I and Figure 7E–I show that TADs delineate regulation of gene expression at their borders and concentrations of genic and regulatory features, as well as nFGFR1 binding, contribute to their formations.

While the multi-TAD islands are dynamically remodeled during cell development, genes of related ontogenic functions, regardless of where they are positioned in the linear genome, are transiently incorporated into the 3D islands allowing their concerted expression. The islands become hubs of genomic subroutines that underwrite different stages of the cell’s development. Table 1 points to complex roles of TFs in the formation of chromatin TADs. Although several TFs appear to contribute to the baseline (ground level), cell stage independent, chromatin structures the pluripotency TFs contribute specifically to TADs that group the ESC highly expressed genes that become inhibited during differentiation. In contrast, TFs known to control neuronal development delineate TADs that group the NCC activated genes.

Figure 6J–L and Figure 7J–L show that within TADs, internal looping reorganizes during ESC to NCC development, and that CTCF associates predominantly with the ESC looping and nFGFR1 with the NCC looping. Our results promote a paradigm in which the ESC TAD islands are formed by CTCF in a process coordinated with nFGFR1. During ESC differentiation, the accumulating nFGFR1 is engaged in the deconstruction of the ESC TADs and in the formation of new TAD islands in developing neurons. In addition to the direct TADs targeting, nFGFR1 may engage in the reconstruction of TAD islands through its inhibition of genes that encode pluripotency TFs and activation of genes that encode neural development promoting TFs [2] (Figure 10D).

In agreement with the genomic analyses, microscopy reveals CTCF and FGFR1 at closely positioned nuclear loci in ESC and in NCC, and nFGFR1 accumulation at distinct nuclear domains in NCC. These observations suggest a nuclear context for the genome archipelago model. The relation between TADs islands and nFGFR1 and CTCF rich nuclear sites requires further investigation. Likewise, the illumination of how the aberrant function of nFGFR1 in cancer cells [12,17,21] may affect genome structural programing could offer a new perspective on cancer.

Together, the roles of nFGFR1 in the archipelago model are based on number of observations: (1) nFGFR1 is concentrated at the borders of TADs during NCC differentiation across the genome, (2) in the Hox clusters, binding of nFGFR1 increases at the promoters of genes during their NCC upregulation and chromatin reorganization, (3) using the FGFR1 blocker, PD137034, nFGFR1 reduction is shown to inhibit normal loop formations within the exemplary HoxA locus, and (4) machine learning shows that the binding of FGFR1 is predictive for the DNA interactions similar as for the gene activities. Through these actions, we propose that nFGFR1 may participate in TAD formations. We have previously shown that nFGFR1 acts as a global regulator of ontogenic groups of genes (neural development, pluripotency, Hox) [2], and the current results support an additional proposed role of nFGFR1 as a global TAD regulator. Additionally, nFGFR1 could play similar roles in the human genome in which FGFR1 binding targets HoxA-D genes and other genes, analogous to its activities in mouse cells [4].

The proposed archipelago model helps to explain how functionally related genes that change chromosomal locations during evolution may maintain co-regulation across diverse evolutionary groups and species. The present investigation reveals conserved protein binding sequences that engage interactions in ESC and which are different in NCC. The illumination of their roles in genome structure, remodeling, and functional programming offers a rousing path towards understanding genome programmatic evolution and its aberration in cancer cells.

Further progress including an expanded resolution to the interactomes, will open greater yet insight into the interactive microdomains, the interactive loops that link specific genes, and the mechanisms of their formation.

## 4. Materials and Methods

### 4.1. Experimental Design

Feeder cell independent E14Tg2A mouse ESCs were grown in L-Glutamine, 4.5 g/L Glucose, and Sodium Pyruvate Dulbecco’s Modified Eagle Medium (DMEM) (10-013-CV Corning, Corning, NY, USA) containing 10% Fetal Bovine Serum (SH30070.03HI GE Healthcare, Chicago, IL, USA), 1% non-essential amino acids, 1% Pen/Strep, 0.06 mM β-mercaptoethanol, 100 U/mL Leukemia Inhibitory Factor (ESG1106 Millipore, Burlington, MA, USA). Cells were maintained in a water-saturated atmosphere at 37 °C containing 5% CO2. Cells to be harvested for experiments were split in the evening, then grown overnight and the next morning were washed with 37 °C Dulbecco’s phosphate-buffered saline (DPBS) to remove LIF and new media was added containing either 100 U/mL LIF, 1 μM Retinoic Acid (R2625 Sigma-Aldrich St. Louis, MO, USA), 100 U/mL LIF plus 10 nM PD173074 (PD) (ab141117 Abcam, Cambridge, MA, USA), or 1 μM Retinoic Acid plus 10 nM PD and grown for 48 h with a change in media completed at 24 h of exposure. Cells were harvested at densities of 60–80%. LIF was supplemented to cell cultures to maintain cells in the ESC condition and Retinoic Acid (RA) was supplemented to media to initiate nFGFR1 influx into the nucleus and cellular differentiation from ESC to NCC [1]. PD, a potent FGFR1 kinase inhibitor which impedes FGFR1 nuclear accumulation [44], was administered to the media in order deplete nFGFR1 in ESC or to diminish the RA-induced nuclear nFGFR1 accumulation in NCC.

### 4.2. ChIP-seq, RNA-seq, HiC, and HiChIP Datasets

FGFR1 binding site peak locations and RNA-seq gene expression levels were incorporated into the current analysis from work previously completed in our lab described in Terranova et al. 2015. RNA-seq raw reads were processed using the Tophat/Cufflinks/Cuffm erge/ Cuffdiff Pipeline [60]. nFGFR1 narrow peak binding strength analysis was completed using the Bowtie 1.0 and MACS2 processing pipeline [2]. Narrow Peak format nFGFR1 ChIP-seq, and RNA-seq FPKM ESC and NCC files for this study can be found at NCBI geo accession number GSE65698 [2]. Three ESC and NCC RNA-seq datasets (GSM1603282, GSM1603283, GSM1603284, GSM1603285, GSM1603286, GSM1603287), and the GSM1603268 and GSM160 3275 nFGFR1 ChIP-seq datasets were used in this study.

The Hi-C/HiChIP protocols were prepared following publications by Mumbach et al. 2016 [30], and Rao et al. 2014 [7] with minor alterations. The Hi-C and HiChIP samples were sequenced from two biological replicates using sample multiplexing (Table S1 Sheet (1) on an Illumina NextSeq 500 with a read length of 75 bp. Global analysis of HiC-Pro and Juicer processed reads of the duplicate samples show their similarity at developmental associated loci. HoxA-D clusters show comparable organization between replicates for both ESC and NCC HiC data (Arrow points to the NCC reorganized HoxA cluster in Figure S1A), so the replicates were merged together for subsequent analysis steps. The two combined biological replicates yielded approximately 170–180 million PETs for Hi-C and 50–70 million PETs for HiChiP. HiC-Pro processing identified approximately 62–68 million valid interaction pair reads per condition for Hi-C and 14–24 million for HiChIP, which were subsequently normalized between compared cell type conditions (ESC and NCC) (Table S1 Sheet 2).

To assess the quality and to overview the ESC and NCC interactomes, we applied Juicer processing [27] to the HiC-Pro output filtered valid reads. The resulting interaction patterns were analyzed in comparison to randomized interactions generated by shuffling the Anchor 1 and Anchor 2 connection locations for each chromosome separately.

As an additional control we used the “expected control” available within the Juicer software. This control developed by Rao in 2014 predicts the expected interaction structure based on the read density and the assumption that increasing distance between loci decreases contact frequencies.

### 4.3. Hi-C/HiChIP Data Processing and Combined Analyses

#### 4.3.1. Identifying Chromatin Loops, Interaction Anchor Strength, and TADs

Pre-Processing (de-duplication of reads, identifying valid reads) was completed using Hi-C Pro [61] using the default settings. The number of valid pairs used in the analyses were normalized between the cell type conditions when comparing samples. Binning of Hi-C Pro output interaction data to quantify chromatin looping was completed using hichipper [62] with the default settings, with each sample analyzed individually and all interaction connection types included in the analysis (F-F/F-R/R-R/R-F) (EACH ALL settings) and additional processing steps were completed using R data processing libraries [63]. Interaction looping score outputs by hichipper were also used for calculating interaction anchor strength by summing up the interaction scores which overlap a location, regardless of which of the two anchors of the interaction the score is from. Filtering by the False Discovery Rate (q) method included in the hichipper software was not used for the main figures, so to not remove the distributions around peaks for calculations, but an example of filtering by q < 0.001 for interaction anchor strength is shown in Figure S7A.

Low copy repeats (LCRs), or segmental duplications, typically 10–300 kb in length, possess 95% sequence identity. Although LCRs occupy a significant part of the human genome, owing to large expansion during primate evolution [64], they are rare in most mammals. Furthermore, the average 75 nt reads generated in our study which do not map to the latest mouse genome build, will be disregarded, even if one side maps to a chromosomal region. However, larger LCR may not be discarded and thus, conceivably could be included within large numbers of interchromosomal and long-distance intrachromosomal interactions detected in our HiC analyses. Further experimental validation using 3C, and/or, fluorescent in situ hybridization (FISH) will shed light on this common matter. In this regard, the HiC detected interactions between Hox loci (Figure 8, Figure S14) are corroborated by earlier FISH studies, [65] and the interactions within the HoxA locus by our 3C analysis (Figure 9).

Comparisons of Interaction scores with other genomic attributes (ChIP-seq, RNA-seq, Genomic Annotations) were completed using the genomic and data processing tools available in R version 3.5.1 [63]. To identify TAD locations, raw Hi-C paired-end fastq files were processed using HiCtool [29], a tool for identifying TAD occurrences using the calculation for TAD identification by Dixon et al. 2012 [6]. TAD identification examples are shown in Figure 3, and the total number and attributes of the TADs found are shown in Figure S2B.

#### 4.3.2. Visualization of Data Analyses

Data was visualized using several graphical software programs. Circos [66] was used for generating circle diagrams. R-Sushi was used for showing chromatin looping on genomic spans [67]. R-igraph was used for plotting ring graphs [68]. Juicer software was used for processing and graphing contact map data. The markers output by the juicer software were enlarged using GNU Image Manipulation Program (GIMP) software. R-ggplot2 and R-plot were used for generating line, point, histogram, and tile graphs. qPCR analysis results were visualized using GraphPad Prism.

#### 4.3.3. Uniform Comparison Matrix-1 kb Binned Genome and Other Binning

Following the RNA-seq, ChIP-seq, HiC, and HiChIP processing pipelines described above, the datasets were overlaid across 1 kb genomic bins so that comparisons could be calculated across uniform genomic ranges. For Principal Component Analysis (PCA) and for location analyses of genomic features, the datasets were combined into a single 1 kb binned genome dataset. Using the mm10 chromosome size limits, every 1000 bp range was made a separate row and each genomic feature being compared was assigned as a separate column. Using the R-GenomicRanges package [69], overlapping locations between the 1 kb binned genome and the genome features of interest (Hi-C interaction anchor strength, ChIP-seq binding scores, RNA-seq gene expression FPKM, and genome structural features from the R-annotatr package [26] for Intergenic, Inter-CpG, lncRNA, Enhancers, Exons, Intron-Exon Boundaries, Introns, Exon-Intron Boundaries, First Exons, 3UTRs, Promoters, Coding Sequences (CDS), 1 to 5 kb Upstream of TSS, CpG Shelves, CpG Shores, CpG Islands, and 5UTRs) were aggregated into the dataset. For features which contained a score (Hi-C interaction anchor strength, ChIP-seq, RNAseq), the score was summed up for the overlapping locations. For features which were only a location (e.g., Exon or Intron locations and other features from the R-annotatr package), the number of base pairs which spanned the 1000 bp region where summed up for its value. A limit of 1000 bp per 1000 bp bin was set for R-annotatr features, to limit features that can be counted multiple times at a location due to their activity with multiple different genes (enhancers and promoters for example) (Figure S2F).

For this study data was binned across genomic ranges and statistics were calculated between the different bins. PCA was completed in for the data in 1 kb bins from the 1 kb binned genome dataset. The bin size was expanded to 5 kb bins for figures showing specific genome locations and averaged together loci. The motif enrichment analysis was completed in 10 kb bins.

#### 4.3.4. All against All Binned Interactions Full Genome Paired *t*-Tests

To visualize the main chromatin interaction differences between ESC and NCC, paired *t*-tests were completed for identifying differences for interactions at every location in the genome versus every other location in the genome in 1 mb × 1 mb bins. Intra- and Inter- chromosomal post hichipper interaction data was binned across a 100 kb × 100 kb binned genome scaffold using R-GenomicRanges [69]. Following the 100 kb binning a second binning step was completed which bins the 100 kb × 100 kb bins into 1 mb × 1mb bins, and this results in the 1 mb bin combinations containing all the combinations of 100 kb bins that occur within (100 values from 100 kb bin interaction combinations within each 1 mb bin combination). Paired *t*-tests were completed comparing ESC to NCC and locations of *p* < 0.05 thresholded differences were plotted in Circos. The coordinate maps were produced with each row being a plot of locations of *p* < 0.05 thresholded differences between two chromosomes (Chr A: 0—Max bp on X-axis, Chr B: 0—Max bp on Y-axis) with the points being locations where the interactions are more common in ESC (blue) or NCC (red). The glyph size of the points scales with p, with smaller *p* values shown as larger glyphs (Figure S3A,B). With approximately 4 million comparisons completed, the Bonferroni correction was not appropriate here since it can be considered as too conservative for tests on large numbers of comparisons [70]. To evaluate the differences between ESC and NCC we used network theory based analyses. The analyses were completed on the top 5000 ESC or NCC 1 mb × 1 mb interaction bins, omitting average strength >1 interactions (a 1 mb × 1 mb bin value is from 100 values averaged together) so that 5000 locations can be viewed together in the same scale range. The ring networks were generated for ESC intra stronger interactions, NCC intra stronger interactions, ESC inter stronger interactions, and NCC inter stronger interactions (Figure S3C). Analyses were completed to show mean percent increases and decreases in interaction strengths, and differences in clustering coefficients and node degree distributions using the Network Analyzer tool available in Cytoscape [28].

#### 4.3.5. Location Specific Binned Interactions Paired *t*-Tests

To investigate the chromatin looping changes at specific loci, at the Hox clusters in this study, the same methodology described above for full genome binned paired *t*-test analysis was completed in +/−1 mb ranges surrounding the Hox A/B/C/D clusters using 1 kb × 1 kb bins within 10 kb × 10 kb bins for the calculations. The X-axes are zero centered on the midpoints of the span of each of the clusters (Chr6:52,208,196, Chr11:96,281,286, Chr15:102,978,977, Chr2:74,716,726). The results are shown as differential loops in which only the stronger (positive delta change values) are shown for each condition, with paired *t*-test statistics with Bonferroni correction shown above the midpoints of the loops, *p* < 10^−5^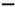; *p* < 10^−15^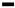; *p* < 10^−25^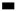 (Figure 9A,B and Figure S15E,F) (Statistics are shown only for one loop, the strongest delta change, per midpoint for better visualization clarity). Log2 fold change FPKM, nFGFR1 binding, and Delta Change Interaction Strength values from the 1 kb binned genome data structure are shown with the chromatin looping changes to show the relationship between chromatin structure changes, gene expression changes, nFGFR1 binding, and interaction anchor site changes (Figure 9C–H and Figure S15G–L).

#### 4.3.6. Alignment Comparisons

For analysis of TADs of the same size, 200 TADs of 480 kb width were aligned together (Figure S2C). The TADs were then sorted by their average interaction anchor strength score throughout their entire TAD range. With the TAD order still sorted by interaction anchor strength, means of other genomic features (RNA-seq FPKM, nFGFR1 ChIP-seq binding, 5UTRs, CpG Islands, Promoters, and Intergenic) were calculated to determine which attributes sorted together with interaction anchor strength inside TADs. A heatmap analysis graph is used to show the intensity or occurrence of the attribute being described within each TAD. Means of the rows (Row means-5 TADs per data point) were calculated to quantify the occurrence of attributes in higher and lower interaction strength containing TADs. Pearson’s R and R2 values show calculated correlation values between interaction strength and the other attributes. The aligned TAD heatmap graphs are marked with ESC TADs (in blue) or NCC TADs (in red) to designate which condition the locations occur from (Figure 4 and Figure S5). Statistics were completed using Z-Score Statistics: Two-Tailed Probability from the Central Area, *p* < 0.05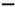; *p* < 0.01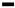; *p* < 0.005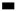.

#### 4.3.7. PCA Analysis

Principal Component Analysis (PCA) was completed on the 1 kb binned genome to identify correlations between Structural (Hi-C), Functional (RNA-seq FPKM), Mechanistic (nFGFR1 ChIP-seq Binding), and Genomic Annotation attributes (Intergenic, lncRNA, Enhancers, Exons, Intron-Exon Boundaries, Introns, Exon-Intron Boundaries, First Exons, 3UTRs, Promoters, Coding Sequences (CDS), 1 to 5 kb Upstream of TSS, CpG Shelves, CpG Shores, CpG Islands, Inter-CpG, and 5UTRs). The angles between the attributes indicate the degree of likelihood that the features correlate together. Each PCA was completed using all rows (all 1 kb genomic location bins) from the 1 kb binned genome datasets, with the Hi-C, RNA, and nFGFR1 ChIP-seq values correlated against the Genomic Annotation attributes separately for ESC and NCC (Figure 2A,B).

#### 4.3.8. Machine Learning

Machine learning was completed on the combined 1 kb binned genome datasets to assess the predictability of RNA FPKM by other genomic attributes. A deep neural network two-window prediction model was used containing 7 layers with 64, 24, 24, 12, 12, 10, and 8 nodes respectively. Each node used the ReLU activation function. The genome locations were randomized and split into training and testing data groups. Machine learning was then applied to train the model on the training data and test its accuracy on unseen testing data. Our deep neural network employed 2 or 3 output nodes (using the SoftMax activation function) depending on whether two or three different FPKM output clusters were used to predict from. We used a sliding window algorithm that calculates the concentrations of the inner and outer genomic regions of each input and uses that calculation as input data. The results show the accuracies that our neural network was able to obtain for each input, or group of inputs, for each range of locales (Figure 2C–F). When predicting interaction score, we used two output classes based on whether the interaction score value is below or above the average interaction score and a single 150/220 sliding window (Figure S4).

#### 4.3.9. All TADs Analysis

For analysis of all the TADs at the same time, all the HiCtool identified TADs were split in half and aligned separately by their left and right boundaries, leaving a gap in the middle for TADs that are shorter than the longer TADs (Figure S2D). A heatmap is used to show the intensity or occurrence of the attribute described within each graph. Means of the heatmap columns (Column means: 5–1 kb bins per data point) were calculated to quantify the occurrence of attributes on the borders and inside TADs. Means of the heatmap rows (Row means: 5 TAD bins per data point) were calculated to quantify the occurrence of attributes in smaller and larger TADs (Figure 5A–H and Figure S6A–N). Statistics were completed using Z-Score Statistics: Two-Tailed Probability from the Central Area, *p* < 0.05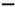; *p* < 0.01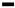; *p* < 0.005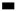.

#### 4.3.10. Regulated Genes-Chromatin Structure Analysis

To identify chromatin loops which are involved in bringing together interacting upregulated (or downregulated) genes across the genome, the HiC-Pro and hichipper processed interaction datasets were annotated at the anchor 1 and anchor 2 genome locations of each interaction pair with log2 fold change RNA-seq expression levels between ESC and NCC data [2]. The interactions were filtered by hichipper q < 0.001 to select high confidence chromatin loops. The interaction loops were ranked in a list from the highest to the lowest sum of RNA-seq log2 fold change FPKM present in the anchor 1 plus the anchor 2 ends of the loops. The top 200 interacting upregulated (or downregulated) gene locations following RA induced NCC differentiation were aligned by their interaction midpoint locations, and then aligned by there overlapping TADs for the later DiffTAD analyses (400 locations for DiffTAD analysis is shown in Figure S7B). These top 200 interacting upregulated (or downregulated) gene aligned midpoints were used to compare location relative NCC/ESC RNA-seq log2 fold changes in gene expression, identify their TAD domain boundaries, and calculate the corresponding interaction directionality changes in +/−1 mb ranges (Figure 6A–C and Figure 7A–C). The data was averaged (RNA-seq and interaction directionality) or summed (number of overlapping TAD Domains) within 5 kb bins. The statistics were completed using the Two-way ANOVA Tukey Method: ESC versus NCC grouped in 5 kb bins, *p* < 1^−2^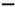; *p* < 1^−4^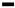; *p* < 1^−6^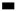; *p* < 1^−8^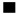; *p* < 1^−10^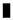.

#### 4.3.11. Regulated Genes-Gene Ontology Analysis

The top 200 interacting upregulated (or downregulated) gene midpoint +/−1 mb regions were analyzed for Gene Ontology (GO) overrepresentation. For each 1 kb bin, the (GO category count/# genes) was zero-centered by the average (GO category count/# genes) for the entire +/−1 mb analyzed genomic span to identify regions of an increased occurrence of GO categories. GO categories for proliferation, general metabolic, developmental, transcriptional regulation, and neuronal gene functions are shown by their relative overrepresentation in +/−1 mb of NCC upregulated and downregulated gene regions (Figure 6D and Figure 7D and S7C). Statistics were completed on the occurrence of GO categories using values from all 10 of the GO categories shown in each figure averaged together within 5 bin spans (5 kb) using Z-Score Statistics: Two-Tailed Probability from the Central Area, *p* < 0.05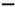; *p* < 0.01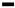; *p* < 0.005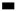.

#### 4.3.12. Regulated Genes—Aligned TAD Analysis

The top 200 interacting upregulated (or downregulated) gene midpoint +/−1 mb regions were analyzed by taking the TADs which overlapped the centered midpoint from each of the top 200 midpoint locations and aligning their left and right borders left (5’) and right (3’) (left-side panel graphs and right-side panel graphs). TADs were aligned based on the average directionality span (per kb) of the significantly upregulated (or downregulated) genes (q < 0.05) which occur within them, NCC downregulated genes for downregulated graphs and NCC upregulated genes for upregulated graphs (Figure 6E–I and Figure 7E–I). The first graph row shows the individual TADs sorted from shortest to largest so changes between ESC and NCC of TADs overlapping the Diffgene midpoints can be seen. The next three graph rows show the changes between the ESC and NCC conditions using three different metrics, Log2 fold change RNA FPKM, Delta Change Interaction Anchor Strength, and Delta Change nFGFR1 binding, with the color indicating if the feature described is stronger in the ESC (blue) or NCC (red) condition at each assessed location. The last row shows a cumulative plot of the average occurrence of genic and regulatory genomic features (Exons, Promoters, CpG Islands, and 5’ UTRs). For these quantitative analyses, values past the TAD midpoints (and therefore closer to the other border than the aligned ones) were omitted from the calculations to prevent features from the left and right sides of TADs borders from averaging together with each other. For these graph sets binning was completed using 5 bins (5 kb spans) per data point and the statistics were completed using Z-Score Statistics: Two-Tailed Probability from the Central Area, *p* < 0.05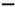; *p* < 0.01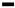; *p* < 0.005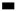.

#### 4.3.13. Regulated Genes—Aligned TAD Analysis—Motifs

Using the top 200 upregulated (or downregulated) gene regions aligned by their left and right TAD borders described above, motif overrepresentation was calculated within 10 kb bins +/−195 kb from both the left and right TAD borders. 50 iterations of 10 repeats of 1000 samplings of 100 bp taken at random within each 10 kb bin was completed to calculate motif overrepresentation using the motif discovery software R-GADEM [24] and R-MotIV [25] for upregulated (or downregulated) gene containing NCC TADs. For locations inside the aligned TADs, locations past the TAD midpoints (and therefore closer to the other border than the aligned ones) were not included for sampling to prevent features from the left and right sides of TADs borders from averaging together with each other (Figures S8–S11). Motifs which were only counted 10 or less times throughout the iterations were omitted from the calculations to reduce false positives. Statistics were completed using Z-Score Statistics: Two-Tailed Probability from the Central Area, *p* < 0.05: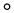; *p* < 0.01: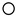; *p* < 0.005: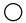. Additionally, Bonferroni correction was applied to the Z-Score derived *p* values based on the number of proteins analyzed to mark the highest confidence motifs in each location adjusted *p* < 0.05: +.

Exemplary motifs of interests are shown using a facet grid graph setup in Figure S12. Same as with the all motifs view, statistics were completed using Z-Score Statistics: Two-Tailed Probability from the Central Area, *p* < 0.05: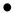; *p* < 0.01: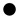; *p* < 0.005: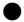; and Bonferroni correction was applied to the Z-Score derived *p* values based on the number of proteins analyzed to mark the highest confidence motifs in each location adjusted *p* < 0.05: +. In addition, 500,000 samplings of 20 bp sequences in each 10 kb bin, +/−195 kb surrounding the ESC and NCC DiffTAD borders, we re taken to determine short DNA sequences contributing to regulated TAD formations. Table S1 Sheets 7–10 shows sequences overrepresented using Z-Score Statistics: Two-Tailed Probability from the Central area, filtered by Bonferroni adjusted *p* < 0.05.

For analysis of nFGFR1 targeted motifs, in the top 200 upregulated (or downregulated) gene regions, nFGFR1 ChIP-seq narrow peak binding sites were analyzed + −100 kb surrounding each aligned TAD border by R-GADEM and R-MotIV motif analysis. The motif discovery calculations were completed 10 times for each 200 kb bin, with the peaks read into the program in a random order with each of the 10 runs to reduce calculation bias, and 10 iterations of those steps were averaged together, before calculating Motif Occurrence as described above. nFGFR1 Binding Preference was calculated from Motif Occurrence, calculated as; the number of times each motif occurred in comparison to the other identified motifs, and is calculated by the mean count of each motif as a part of a 1.0 whole of all the nFGFR1 bound motif counts added together (Figure S13). nFGFR1 binding has a strong consistency for which motifs it binds to at both upregulated and downregulated NCC TAD borders so Z-Score Statistics did not find location specific differences between different 200 kb bins for nFGFR1 binding affinity.

For the combined tables of the motif results, the motifs which occurred as *p* < 0.05 overrepresented at least once within +/−35 kb of the NCC− or NCC+ TAD borders are shown on the table. The lowest *p* value within the +/−35 kb range for each border is shown. The table is sorted by motifs enriched in all 4 borders, motifs enriched in 3 borders, motifs enriched in 2 borders, and motifs enriched in 1 border (Table 1). Bonferroni adjusted *p* < 0.05 are marked with a + and are highlighted. All motifs which were identified as targeted by nFGFR1 in ESC or NCC (regardless of relative targeting strength) are marked by a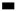.

#### 4.3.14. Regulated Gene Containing TADs Differential Looping

To investigate the main chromatin looping changes at the top 200 interacting upregulated (or downregulated) gene containing TADs, the same methodology described above for full genome binned paired *t*-test analysis was completed in +/−500 kb ranges surrounding the left and right aligned upregulated (or downregulated) gene containing NCC TAD borders, with the X-axes origins centered on the aligned TAD borders using 1 kb × 1 kb bins within 10 kb × 10 kb bins for the calculations. Loops that ended past the midpoint of TADs were removed from the calculation so that the left and right aligned borders features would not show attributes from both ends of the TADs mixed together. The results are shown as differential loops in which only the stronger (positive delta change values) are shown for each condition, with paired *t*-test statistics with Bonferroni correction shown above the midpoints of the loops, adjusted *p* < 10^−5^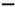; *p* < 10^−15^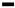; *p* < 10^−25^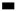. (Statistics are shown only for one loop, the strongest delta change, per midpoint for better visualization clarity). Hi-C chromatin looping, nFGFR1 chromatin looping, and CTCF chromatin looping are shown together to show the main changes in chromatin organization in differential gene expression containing TAD borders, and how nFGFR1 and CTCF are involved in the chromatin looping changes (Figure 6J–L and Figure 7J–L ).

### 4.4. 3C-qPCR, ChIP-qPCR, and RT-qPCR Sample Preparations and Quantification

Chromosome Conformation Capture (3C) samples were prepared following methods previously described in [5,71] with minor changes using Hind III for restriction digestion (10798983001 Roche, Branchburg, NJ, USA). Chromatin Immunoprecipitation (ChIP) samples were collected and prepared using the MAGnify Chromatin Immunoprecipitation System Kit (492024 ThermoFisher, Waltham, MA, USA) with minor alterations. Immunopreciptations were completed using FGFR1 ab10646 Abcam, Cambridge, MA, CTCF ab70303 Abcam, Cambridge, MA, USA and control Rb IgG MAGnify 492024 ThermoFisher, Waltham, MA, USA. Samples for mRNA were collected using the RNeasy Mini Kit (74104 Qiagen, Germantown, MD, USA) with minor alterations. Primer designs are shown in Table S1 Sheet 3. Three technical replicates were completed on the same plate for each biological replicate measurement. Three biological replicates were completed for 3C-qPCR, five for FGFR1 ChIP-qPCR, three for CTCF ChIP-qPCR, and three for RT-qPCR.

3C-qPCRs measurements were calculated by Percent Input of a non-digested location

(at the GapDH gene):

Percent Input = [100 * 2⌃(GapDH CT − Interaction CT)] * 100

ChIP-qPCR measurements were calculated as Percent 10% Input:

Percent Input = 100 * 2⌃(10% Input CT − IP sample CT)

RT-qPCR measurement were calculated as Relative Expression:

Relative Expression = [100 * 2⌃(−1 * cDNA CT)] * 10^6^

### 4.5. Immunocytochemistry and Microscopy

Double immunostaining was performed according to our established protocol [3,23] using mouse αFGFR1 (Novus Biologicals, Centennial, CO, 1:200) and rabbit αCTCF (ab84372, 1:500, Abcam, Cambridge, MA, USA). As the secondary antibodies we used anti-mouse Alexa 568 and anti-rabbit Alexa 488. Specificity of immunostaining was ascertained with control reactions in which the primary antibody was omitted or replaced with pre-immune sera, or by neutralizing the antibody with cognate peptide. Staining was observed using a Nikon Diaphot microscope or Bio-Rad MRC 1024 confocal microscope (BioRad Laboratories, Hercules, CA, USA). The possibility of bleed-through in double fluorescent cells was excluded by acquiring images in sequential mode.

### 4.6. Statistical Analysis

Statistical approaches were used throughout this study to analyze bins versus other bins in histograms (Z-Score Statistics), ESC histograms versus NCC histograms at the same bins (2-Way ANOVA Tukey), and interaction looping differences between ESC and NCC (Paired *t*-test). Z-Score Statistics: Two-Tailed Probability from the Central Area was used in assays to identify genomic locations (bins) within the ranges specified (e.g., +/−1 mb spans) in which the attribute score being analyzed falls outside the normal distribution of the scores for the entire range. Paired *t*-tests were used to compare binned interaction scores between ESC and NCC conditions using the *t*-test function available in the R-stats library, and where specified, the values were corrected using the Bonferroni method for multiple comparisons to adjust *p* values based on the number of tests performed. Two-Way ANOVA Tukey Method was completed to compare ESC and NCC values for RNA, TAD coverage, and interaction directionality analyses. Each test was completed on 5–1 kb values of ESC versus 5–1 kb values for NCC using the ANOVA and Tukey Method functions available in R-stats. Two-Way ANOVA with Fisher’s LSD Test was used to determine significance for the qPCR results for the HoxA cluster ESC versus NCC and PD inhibition analyses using the tools available in GraphPad Prism software.

## Figures and Tables

**Table 1:** Regulated TADs are under control of distinct transcription factors and architectural proteins during ESC to NCC differentiation. +/−35 kb border enrichment of protein binding motifs and +/−100 kb enrichment of nFGFR1 border motif targeting. Paired *T*-Test, Bonferroni adjusted *p* < 0.05 highlighted +.

		NCC−	NCC−	NCC+	NCC+	ESC	NCC			NCC−	NCC−	NCC+	NCC+	ESC	NCC
#	Motif	5’	3’	5’	3’	FGFR1	FGFR1	#	Motif	5’	3’	5’	3’	FGFR1	FGFR1
**1**	**CTCF**	*p* = 0.000419 +	*p* = 0.016	*p* = 0.0000406 +	*p* = 0.000155 +			**53**	**E2F1**	*p* = 0.0000996 +			*p* = 0.00389	⏹	
**2**	**MYC..MAX**	*p* = 0.00319	*p* = 0.000136 +	*p* = 0.0000245 +	*p* = 0.00282	⏹		**54**	**Foxa2**	*p* = 0.00224			*p* = 0.00302		
**3**	**NFIC**	*p* = 0.0472	*p* = 0.02	*p* = 0.0286	*p* = 0.00977			**55**	**FOXO3**	*p* = 0.0227			*p* = 0.0302		
**4**	**NFKB1**	*p* = 0.00632	*p* = 0.0284	*p* = 0.0252	*p* = 0.0309			**56**	**Nkx2.5**	*p* = 0.000182 +			*p* = 0.0391		
**5**	**Pdx1**	*p* = 0.0299	*p* = 0.0415	*p* = 0.0175	*p* = 0.000532			**57**	**SRY**	*p* = 0.0259			*p* = 0.0192		
**6**	**Spz1**	*p* = 0.0193	*p* = 0.0059	*p* = 0.0327	*p* = 0.00452			**58**	**ESR1**	*p* = 0.0274		*p* = 0.0311			
**7**	**ZEB1**	*p* = 0.00383	*p* = 0.0284	*p* = 0.00849	*p* = 0.0178	⏹		**59**	**FOXD1**	*p* = 0.0368		*p* = 0.0465			
**8**	**ARID3A**		*p* = 0.0343	*p* = 0.0114	*p* = 0.012			**60**	**HNF1B**	*p* = 0.000173 +		*p* = 0.00459			
**9**	**Mycn**		*p* = 0.0126	*p* = 0.0493	*p* = 0.000000141 +			**61**	**MEF2A**	*p* = 0.000206 +		*p* = 0.00314			
**10**	**PLAG1**		*p* = 0.0278	*p* = 0.0181	*p* = 0.00189	⏹		**62**	**PPARG**	*p* = 0.00667		*p* = 0.00881			
**11**	**Ddit3..Cebpa**	*p* = 0.0446		*p* = 0.0394	*p* = 0.000392 +			**63**	**PPARG..RXRA**	*p* = 0.0023		*p* = 0.0238		⏹	⏹
**12**	**ELK4**	*p* = 0.0359		*p* = 0.0026	*p* = 0.014			**64**	**SOX9**	*p* = 0.00105		*p* = 0.0000956 +			
**13**	**Foxq1**	*p* = 0.00347		*p* = 0.0172	*p* = 0.0045			**65**	**SP1**	*p* = 0.0409		*p* = 0.0304		⏹	⏹
**14**	**HNF4A**	*p* = 0.00214		*p* = 0.0263	*p* = 0.045			**66**	**TEAD1**	*p* = 0.0228		*p* = 0.000765		⏹	⏹
**15**	**NR1H2..RXRA**	*p* = 0.00637		*p* = 0.00468	*p* = 0.0199			**67**	**Zfp423**	*p* = 0.0077		*p* = 0.0218		⏹	
**16**	**NR4A2**	*p* = 0.0227		*p* = 0.00945	*p* = 0.0194			**68**	**GATA2**	*p* = 0.0417	*p* = 0.0358				
**17**	**RXRA..VDR**	*p* = 0.000555		*p* = 0.0217	*p* = 0.00115			**69**	**MAX**	*p* = 0.00105	*p* = 0.00151				
**18**	**STAT1**	*p* = 0.0112		*p* = 0.00069	*p* = 0.00509			**70**	**Pax6**	*p* = 0.0327	*p* = 0.0407			⏹	
**19**	**AP1**	*p* = 0.0207	*p* = 0.0125		*p* = 0.0229			**71**	**RELA**	*p* = 0.0199	*p* = 0.0065				
**20**	**Ar**	*p* = 0.00165	*p* = 0.00847		*p* = 0.0004 +			**72**	**SRF**	*p* = 0.0021	*p* = 0.0482				
**21**	**REL**	*p* = 0.0222	*p* = 0.0168		*p* = 0.0356			**73**	**YY1**	*p* = 0.0000987 +	*p* = 0.000421 +				
**22**	**RXR..RAR_DR5**	*p* = 0.016	*p* = 0.0158		*p* = 0.0087			**74**	**FOXI1**				*p* = 0.0234	⏹	
**23**	**TAL1..TCF3**	*p* = 0.000705	*p* = 0.0000833 +		*p* = 0.0165			**75**	**Hand1..Tcfe2a**				*p* = 0.0165		
**24**	**Arnt**	*p* = 0.00146	*p* = 0.0000373 +	*p* = 0.0000167 +		⏹		**76**	**Nr2e3**				*p* = 0.000295 +		
**25**	**BRCA1**	*p* = 0.0182	*p* = 0.0445	*p* = 0.000583		⏹		**77**	**SOX10**				*p* = 0.0436	⏹	
**26**	**ESR2**	*p* = 0.0418	*p* = 0.00483	*p* = 0.00348				**78**	**TFAP2A**				*p* = 0.0396	⏹	
**27**	**FOXA1**	*p* = 0.00129	*p* = 0.0203	*p* = 0.025				**79**	**CREB1**			*p* = 0.00606			
**28**	**GABPA**	*p* = 0.0000218 +	*p* = 0.0184	*p* = 0.0000239 +		⏹		**80**	**Evi1**			*p* = 0.0347			
**29**	**GATA3**	*p* = 0.00391	*p* = 0.00418	*p* = 0.00013 +				**81**	**FEV**			*p* = 0.0112		⏹	
**30**	**HNF1A**	*p* = 0.00000372 +	*p* = 0.00921	*p* = 0.0154				**82**	**FOXF2**			*p* = 0.00178			
**31**	**HOXA5**	*p* = 0.0255	*p* = 0.00488	*p* = 0.0218				**83**	**Mafb**			*p* = 0.000463			
**32**	**INSM1**	*p* = 0.0216	*p* = 0.0215	*p* = 0.0231		⏹	⏹	**84**	**MZF1_5.13**			*p* = 0.00631		⏹	⏹
**33**	**Lhx3**	*p* = 0.000149 +	*p* = 0.0029	*p* = 0.0000534 +				**85**	**NFIL3**			*p* = 0.000132 +			
**34**	**NFE2L2**	*p* = 0.00466	*p* = 0.00185	*p* = 0.000788		⏹		**86**	**NHLH1**			*p* = 0.000326 +		⏹	
**35**	**Prrx2**	*p* = 0.0111	*p* = 0.00459	*p* = 0.00142				**87**	**NR3C1**			*p* = 0.0185			
**36**	**RUNX1**	*p* = 0.0419	*p* = 0.0281	*p* = 0.0000178 +		⏹		**88**	**Pax4**			*p* = 0.0243		⏹	⏹
**37**	**Tal1..Gata1**	*p* = 0.0207	*p* = 0.00127	*p* = 0.0216		⏹	⏹	**89**	**Pax5**			*p* = 0.000701		⏹	⏹
**38**	**Tcfcp2l1**	*p* = 0.0293	*p* = 0.0257	*p* = 0.0397		⏹		**90**	**RORA_1**			*p* = 0.00413			
**39**	**TLX1..NFIC**	*p* = 0.0342	*p* = 0.0015	*p* = 0.0269		⏹		**91**	**RREB1**			*p* = 0.0149		⏹	
**40**	**ZNF354C**	*p* = 0.000762	*p* = 0.0303	*p* = 0.00751		⏹	⏹	**92**	**Sox17**			*p* = 0.00000485 +			
**41**	**En1**			*p* = 0.0004 +	*p* = 0.0447	⏹		**93**	**TP53**			*p* = 0.00666		⏹	
**42**	**Myc**			*p* = 0.000751	*p* = 0.00337	⏹		**94**	**NF.kappaB**		*p* = 0.0106				
**43**	**MZF1_1.4**			*p* = 0.00249	*p* = 0.022	⏹	⏹	**95**	**Pou5f1**		*p* = 0.00291			⏹	
**44**	**Zfx**			*p* = 0.0174	*p* = 0.00635	⏹	⏹	**96**	**CEBPA**	*p* = 0.000467				⏹	
**45**	**Sox5**		*p* = 0.000105 +		*p* = 0.00132			**97**	**Foxd3**	*p* = 0.0281				⏹	
**46**	**Stat3**		*p* = 0.0407		*p* = 0.0454	⏹		**98**	**Klf4**	*p* = 0.0115				⏹	⏹
**47**	**Esrrb**		*p* = 0.0182	*p* = 0.0191				**99**	**NFYA**	*p* = 0.00374					
**48**	**Myf**		*p* = 0.0118	*p* = 0.00443		⏹		**100**	**Nkx3.2**	*p* = 0.0000242 +					
**49**	**NFATC2**		*p* = 0.00737	*p* = 0.0249				**101**	**PBX1**	*p* = 0.00908					
**50**	**REST**		*p* = 0.0238	*p* = 0.00427		⏹		**102**	**Sox2**	*p* = 0.0404				⏹	
**51**	**znf143**		*p* = 0.0421	*p* = 0.0303		⏹		**103**	**T**	*p* = 0.0156				⏹	
**52**	**Arnt..Ahr**	*p* = 0.00464			*p* = 0.0466	⏹									

## Supplementary Materials

Supplementary data to this article can be found online at https://www.mdpi.com/1422-0067/22/1/347/s1. High-Throughput data is available in raw and processed forms at NCBI GEO under accession number GSE153884. Custom R-code used for data processing and graphing analysis is available at https://buffalo.box.com/s/un8frry2liyi60sjkmi9k8jqyadsolet. Other resources are available upon request.

## Abbreviations

The following abbreviations are used in this manuscript:

ESC	Embryonic Stem Cell
NCC	Neuronal Committed Cells
INFS	Integrative Nuclear FGFR1 Signaling
nFGFR1	Nuclear Fibroblast Growth Factor Receptor 1
TAD	Topologically Associating Domains
RA	Retinoic Acid
TF	Transcription Factor
CC	Clustering Coefficients
DD	Degree Distributions
PCA	Principle Component Analysis
Diffgene	Differentially Regulated Interacting Gene
DiffTAD	Differentially Regulated Gene Containing TAD
GO	Gene Ontology
PD	PD173074
3C	Chromosome Conformation Capture
ChIP	Chromatin Immunoprecipitation
