# Supplementary material for: Global Genome Conformational Programming during Neuronal Development Is Associated with CTCF and Nuclear FGFR1—The Genome Archipelago Model
Source: Int J Mol Sci. 2020 Dec 31;22(1):347. doi: 10.3390/ijms22010347 (PMC7795191; doi:10.3390/ijms22010347)
Supplement: Supplementary file 1 [file ijms-22-00347-s001.zip › Supplement_1.docx]

Supplementary Materials

Additional and Expanded Materials and Methods

A. Hi-C / HiChIP Sample Preparation

The Hi-C / HiChIP protocols were prepared following publications by Mumbach et. al. 2016 [30], and Rao et. al. 2014 [7]. Approximately 15 million cells were grown up on 10 cm plates to ~60-80% confluence. An additional plate, for each sample condition, was prepared for cell counting prior to cell collection for normalizing the cell number in the later steps. 37% Formaldehyde was added to each plate to a final concentration of 1% and plates were rotated at room temperature for 10 minutes. 2M glycine was added to a final concentration of 125 mM and the plates were rotated at room temperature for 5 minutes to quench the formaldehyde reaction. The plates were rinsed 3 times with ice-cold DPBS and then 3 ml of DPBS + Protease Inhibitor (PI) cocktail (11697498001 Roche, Branchburg, NJ) was added and the plates were placed on ice. Cells were scraped from each plate into 15 ml plastic conical tubes and were pelleted at 1000 RPM (GS-6R Centrifuge) for 5 minutes. The cell pellets were resuspended in 500 $\mu$l Hi-C lysis buffer (10 mM Tris-HCl pH 7.5 10 mM NaCl, 0.2% Igepal, 1 Roche PI tablet), and the sample volume was normalized based on the extra plate cell count, to reduce the cell number of each sample to 12 million total cells. The samples were rotated for 30 minutes at 80 RPM on ice (American Rotor) to lyse cell membranes and release the nuclei. The nuclei were pelleted by centrifugation at 2500 RCF for 5 minutes, and following aspiration of the supernatant, nuclei were washed (resuspended) in 500 $\mu$l of ice-cold Hi-C lysis buffer. The nuclei were centrifuged at 2500 RCF for five minutes, and after aspirating off the supernatant, were gently resuspended in 100 $\mu$l of 0.5% SDS dH_2_0, until the nuclei were completely homogenous in solution. The samples were incubated at 62 $℃$ for 10 minutes without shaking or rotation, and subsequently on ice for 1 minute, and then 335 $\mu$l of 1.49% Triton-X 100 was added to each sample. The samples were rotated for 15 minutes at 37 $℃$ and 250 RPM (HM100-Pro thermomixer) to quench the SDS with Triton. 50 $\mu$l of NEB Buffer 2 (B7002S New England Biolabs, Ipswich, MA) and 15 $\mu$l of 25 U/ul MboI restriction enzyme (R0147M New England Biolabs, Ipswich, MA) was added to each sample, and the samples were rotated for 2 hours at 37 $℃$ and 900 RPM to restriction digest the DNA within the intact nuclei samples. The samples were placed in a 62 $℃$ water bath for 20 minutes with no shaking or rotation to heat inactivate the MboI enzymes in the samples. The samples were placed on ice for 2 minutes, 52 $\mu$l of Biotin Addition Master Mix was added to each sample (37.5 $\mu$l 0.4 mM Biotin-14-dATP (19524-016 Invitrogen, Carlsbad, CA), 4.5 $\mu$l dCTP, dGTP, and dTTP mix 10mM each (Invitrogen, Carlsbad, CA) (1.5 $\mu$l of each), 10 $\mu$l 5 U/ul DNA Polymerase I, Large Klenow Fragment, M0210S New England Biolabs, Ipswich, MA), and the samples were rotated for 1 hour at 37 $℃$ and 900 RPM to fill in the sticky DNA ends with dCTP, dGTP, dTTP, and biotin labeled ATP. 948 $\mu$l of ligation master mix (150 $\mu$l 10x NEB T4 DNA ligase buffer with 10 mM ATP, 125 $\mu$l 10% Triton-X (B0202S New England Biolabs, Ipswich, MA), 3$\mu$l 50 mg/ml BSA, 10$\mu$l 400 U/uL T4 DNA ligase (M0202L New England Biolabs, Ipswich, MA), 660$\mu$l dH20) was added to each sample, and the samples were rotated end over end at room temperature for 4 hours to generate the proximity ligation connections between the interacting DNA locations. The samples were centrifuged at 2500 RCF for 5 minutes at room temperature to collect intact nuclei. Supernatant was aspirated off and the nuclei pellets were resuspended in 880 $\mu$l Nuclear Lysis Buffer (50 mM Tris-HCl pH 7.5, 10 mM EDTA, 1% SDS, 1 PI tablet). The samples were sonicated using the settings, Output: 2, Duty: 30, 15 intervals, 15 pulses per interval, and 1 minute delays between intervals on a Branson Sonifier 250 to fragment the DNA samples into 150 – 300 base pair fragments. The samples were centrifuged at 16,100 RCF for 15 minutes at 4 $℃$, and then the clarified supernatants were transferred to new 1.5 mL Eppendorf tubes. 16.7% (146.67 $\mu$l, DNA from 2 million cells) of each sample was set aside for Hi-C sample preparations later, while the remainder (DNA from 10 million cells) was split in half for immunoprecipitation (IP) pulldowns using ChIP grade anti-FGFR1 [2] (ab10646 Abcam, Cambridge, MA) or anti-CTCF (ab70303 Abcam, Cambridge, MA) antibodies to generate HiChIP samples. For each HiChIP sample (366.67 $\mu$l), the samples were diluted to a total volume of 1320 $\mu$l with ChIP Dilution Buffer (0.01% SDS, 1.1% Triton X-100, 1.2 mM EDTA, 16.7 mM Tris-HCl pH 7.5, 167 mM NaCl). 30 $\mu$l of Protein A Dynabeads (10001D ThermoFisher, Waltham, MA) were washed with 100 $\mu$l of ChIP dilution buffer, pulled down on a magnetic tube holder, and then resuspended in 100 $\mu$l of ChIP dilution buffer, before being added to each 1320 $\mu$l sample and rotated at 4 $℃$ for 1 hour to preclear the samples before the IP steps. The samples were placed on a magnetic tube holder, and the precleared supernatants were transferred to new sterile tubes. For each 5 million cell sample, 3.75 $\mu$g of antibody was added, and the samples were rotated at 4 $℃$ overnight. The next morning 30 $\mu$l of Protein A dynabeads were washed once and then resuspended in 50 $\mu$l of ChIP dilution buffer and added to each sample. The samples were rotated at 4 $℃$ for 2 hours, before being washed 3x with Low-salt Wash Buffer (0.1% SDS, 1% Triton X-100, 2 mM EDTA, 20 mM Tris-HCl pH 7.5, 150 mM NaCl), High-salt Wash Buffer (0.1% SDS, 1% Triton X-100, 2 mM EDTA, 20 mM Tris-HCl pH 7.5, 500 mM NaCl), and LiCl Wash Buffer (10 mM Tris-HCl pH 7.5, 250 mM LiCl, 1% Igepal, 1% sodium deoxycholate, 1 mM EDTA, make fresh). The samples were spun down, the supernatant was removed, and 150 $\mu$l of freshly prepared DNA Elution Buffer (50 mM sodium bicarbonate pH 8.0, 1% SDS) was added to each sample of beads. The samples were incubated at room temperature with 120 RPM shaking for 10 minutes, and then incubated at 37 $℃$ with 500 RPM shaking for 3 minutes. The eluted samples were collected into separate tubes, and the beads were eluted a second time with 150 $\mu$l of DNA Elution Buffer with 120 RPM shaking at room temperature for 10 minutes, followed by incubation at 37 $℃$ with 900 RPM shaking for 3 minutes. The eluted samples were collected, and combined with the first elutions. At this step Hi-C samples were diluted to 300 $\mu$L with DNA Elution Buffer and processed alongside the HiChIP samples. 15 $\mu$l of Proteinase K was added to each 300 $\mu$l sample, and the samples were incubated at 55 $℃$ for 60 minutes (no shaking for 35 minutes, 500 RPM for 15 minutes, 10 minutes at 900 RPM). The samples were de-crosslinked by incubation at 67 $℃$ for 1 hour 45 minutes (no shaking for 1 hour 15 minutes, 30 minutes at 900 RPM). The DNA from each sample was purified using Zymo Clean and Concentrator Columns (D4013 Zymo Research, Tustin, CA), and eluted into a total volume of 11 $\mu$l molecular grade dH_2_0 (eluted twice using the same 11 $\mu$l volume). The DNA samples were quantified by measuring 1 $\mu$l of sample using the Qubit Fluorometric Assay (Q33216 Qubit 3.0 Fluorometer ThermoFisher, Waltham, MA). Prior to the pulldown steps, 5 ul of Streptavidin C-1 magnetic beads (65001 ThermoFisher, Waltham, MA) per sample were washed with Tween Wash Buffer (5 mM Tris-HCl pH 7.5, 0.5 mM EDTA, 1 M NaCl, 0.05% Tween-20) and then resuspended in 20 $\mu$l of 2x Biotin Binding Buffer (10 mM Tris-HCl pH 7.5, 1 mM EDTA, 2 M NaCl). The DNA samples (2 – 5 ng for HiChIP samples and 80 ng for Hi-C samples) were mixed with the Streptavidin C-1 magnetic beads to bind DNA by their biotin-ATP incorporated nucleotides and were incubated at room temperature with 120 RPM shaking for 15 minutes, pipetted up and down gently, and incubated at room temperature with 120 RPM shaking for another 15 minutes. The beads were washed 2x with 500 $\mu$l of Tween Wash Buffer, washed with 100 $\mu$l of 1x TD Buffer (2x TD Buffer is: 20 mM Tris-HCl pH 7.5, 10 mM magnesium chloride, 20% dimethylformamide), and then resuspended in tn5 Transposase Master Mix (25 $\mu$l 2x TD Buffer, $\mu$l of tn5 Transposase enzyme (FC-121-1031 Illumina, Madison, WI) relative to amount of DNA in sample, fill to 50 $\mu$l with dH_2_0) and incubated at 55 $℃$ with 500 RPM interval shaking for 10 minutes, pausing shaking to resuspend the beads in solution at 8 and 4 minutes during the incubation period. Using a magnetic tube holder, the beads were washed with 50 mM EDTA at 50 $℃$ and 500 RPM shaking for 30 minutes, washed 2x more times with 50 mM EDTA with incubation at 55 $℃$ and 500 RPM shaking for 2 minutes, washed 2x times with 100 $\mu$l of Tween Wash Buffer with incubation at 55 $℃$ and 500 RPM shaking for 2 minutes, and finally washed one time with 10 mM Tris. The buffer was aspirated off the beads and beads from each sample were resuspended in 50 $\mu$l of Phusion PCR Master Mix (25 $\mu$l of NEB Phusion HF 2x (M0531S New England Biolabs, Ipswich, MA), 1 $\mu$l of 12.5 $\mu$M Nextera Multiplex Forward Primer, 1 $\mu$l of 12.5 $\mu$M Nextera Multiplex Reverse Primer, 23 $\mu$l of dH_2_0) (Adapter Primers used are shown in Table S1 Sheet 1). The bead bound DNA was PCR amplified (9 cycles for Hi-C samples, 10 cycles for HiChIP samples – dependent on DNA concentration) using the PCR routine: Initialize – 72 $℃$ for 5 min, 98 $℃$ for 1 min ; Repeat – 98 $℃$ for 15 sec, 63 $℃$ for 30 sec, 72 $℃$ for 1 min. After PCR amplification the samples were purified using Zymo Clean and Concentrator Columns with an elution volume of 10 $\mu$l Zymo Elution Buffer (same 10 $\mu$l put through column twice to increase the concentration of the elution). The purified DNA samples were electrophoresed on a 6% Polyacrylamide Gel at 5 volts for 20 hours. The next day, the polyacrylamide gels were removed from their castes and incubated in Sybr Safe containing TBE buffer (1 $\mu$l Sybr Safe per 100 ml TBE) for 30 minutes, pictures of the gels were taken, and the DNA bands within the 200 – 700 bp range were excised from the gels for each sample. The collected gel fragments were shredded by centrifuging the gels through 200 $\mu$l PCR tubes with a hole poked in the bottom into a 1.5 ml Eppendorf tube. DNA was extracted from the shredded gel samples using the QIAEX II Gel Extraction Kit (20021 Qiagen, Germantown, MD). For the final elution step, DNA samples were eluted into 20 $\mu$l of TE Buffer, and the final library preparation and quality assessment steps (library concentration quantified by Bioanalyzer and PCR analysis) were completed by the University at Buffalo Genomics and Bioinformatics Core. Paired-end sequencing of 75 bp length reads was completed using a NextSeq 550, resulting in ~50 – 70 million reads for HiChIP samples and ~170 – 180 million reads for HiC samples per condition combined together from two biological replicates (Table S1 Sheet 2).

B. Hi-C / HiChIP Data Processing and Combined Analyses

I. Machine Learning

Machine learning was completed on the combined 1 kb binned genome datasets to assess the predictability of RNA FPKM by other genomic attributes. A deep neural network was used containing 7 layers with 64, 24, 24, 12, 12, 10, and 8 nodes respectively. Each node uses the ReLU activation function. Two regions were defined in our analysis: an inner, and an outer region. The inner region is a smaller sector in the input data where values of the input data are summed up. The outer region is a larger sector that covers the inner regions and extend beyond the inner region in both directions, and the values of the entire outer region are summed up. Finally, the inner region summed up value is divided with the outer region summed up value to get the concentration of the input data at each and every location. Individual and grouped sets of attributes were used to determine different ranges of RNA FPKM using this 2-window range (ranges in kb) neural network prediction model. The smaller window contains the attributes used for prediction (FGFR1 binding, interaction strength, genome structural features, or combinations of them) and the larger (or equal to) window predicts the RNA FPKM. The 2-window range method was used to process the input data in a way to overcome the local differences in the sparsity of features throughout the genome and to standardize the raw data based on local maxima and minima regions. The genome locations were randomized and split into training and testing data groups. Machine learning was then applied to train the model on the training data and test its accuracy on unseen testing data. Our deep neural network employed 2 or 3 output nodes (using the SoftMax activation function) depending on whether two or three different FPKM output clusters were used to predict from. The results show the accuracies that our neural network was able to obtain for each input, or group of inputs, for each range of locales (Figure 2C-F).

C. 3C Sample Preparation

Chromosome Conformation Capture (3C) samples were prepared following methods previously described in [5, 71] with minor changes. The cells harvested for 3C analysis were fixed with 37% molecular grade Formaldehyde, which was added to the media to a 1% final concentration and rotated at 40 RPM at room temperature for 10 minutes. 2M glycine was added to media to a 200 mM final concentration and the plates were rotated at 40 RPM for 5 minutes. Each plate was rinsed 3x with ice cold DPBS. 3 mL PBS + PI (11697498001 Roche, Branchburg, NJ) was added to each plate. Cells were scraped into 15 mL conical tubes and centrifuged at 500 RPM for 10 minutes at 4 $℃$. The liquid was aspirated off without disturbing the pellet. The pellet was resuspended in 3C Lysis Buffer (10 mM Tris pH 8.0 10 mM NaCl 0.2% Igepal + PI) and incubated on ice for 10 minutes and then centrifuged at 1000 RCF for 10 minutes. The supernatant was removed without disturbing the nuclei pellet. Each nuclei pellet was resuspended in 1x SureCut Buffer B (11417967001 Roche, Branchburg, NJ) and centrifuged at 1000 RCF for 10 minutes. After removing the supernatant, each nuclei pellet was resuspended in 1% SDS 3x SureCut Buffer B and passed through an insulin needle 10 times to dissociate clumped up cells. The samples were incubated at 65 $℃$ for 10 minutes and then placed on ice for 5 minutes. Triton X-100 and dH20 were added to bring each sample buffer to a final concentration of 0.3% SDS 2% Triton X-100 1x SureCut Buffer B. The samples were incubated at 37 $℃$ for 30 minutes. 333 U of Hind III (10798983001 Roche, Branchburg, NJ) restriction enzyme was added to each sample and the samples were incubated at 37 $℃$ for 1 hour with 900 RPM rotation. 333 U of Hind III restriction enzyme was added a second time to each sample and the samples were incubated at 37 $℃$ overnight with 900 RPM rotation. The next day 333 U of Hind III restriction enzyme was added to each sample and the samples were incubated at 37 $℃$ for 5 hours with 900 RPM rotation. SDS was added to each sample to bring the final SDS concentration to 1.6% SDS. The samples were incubated at 65 $℃$ for 15 minutes to denature the restriction enzymes, and then were placed on ice for 15 minutes. Each sample was diluted with 1% Triton X-100 1x Ligation Buffer (B0202S New England Biolabs, Ipswich, MA) to a concentration of 2.5 ng/$\mu$L of DNA in a final volume of 20 mL. 1800 U of T4 DNA ligase (M0202L New England Biolabs, Ipswich, MA) was added to each sample, which were then rotated for 1 hour at room temperature at 100 RPM. Another 1800 U of T4 DNA ligase was added to each sample and they were rotated overnight at room temperature at 100 RPM. The next day 0.1 mg of RNase A (EN0531 ThermoFisher, Waltham, MA) was added to each sample and the samples were incubated at 37 $℃$ for 30 minutes. 0.2 mg of Proteinase K (EO0491 ThermoFisher, Waltham, MA) was added to each sample and the samples were incubated at 55 $℃$ for 2 hours. The samples were then incubated overnight at 65 $℃$. The next day the samples were phenol chloroform extracted and then ethanol precipitated for DNA purification and DNA from each sample were redissolved in 500 $\mu$l 1x TE Buffer. A second round of phenol chloroform extraction and ethanol precipitation was completed to clean up impurities for each 3C DNA isolation (a second extraction is needed for high quality 3C results). The final DNA pellet was resuspended in 500 $\mu$L of 1x TE buffer and stored in -20 C.

D. 3C-qPCR Quantification

Three technical replicates were completed on the same plate for each measurement of a primer pair for each biological replicate. Three biological replicates were completed for each sample condition. Two forward primers 5’ (NCBI bp numbering notation) to Hind III restriction sites were used for each qPCR interaction measurement, with the HoxA1 forward primer used as an anchor against downstream HoxA cluster members for each interaction measurement. Forward and reverse primers not separated by a Hind III restriction enzyme site were designed for a location on the GapDH gene to be used as control measurement of genomic DNA present in the sample so that the amount of interaction could be calculated as relative to the amount of total DNA present (Figure 9I). The measurements that were used for experimental results for each primer pair were calculated as:

$$Percent Input = [100 *2^(GapDH CT - Interaction CT)] * 100$$

The fold change effect by PD on HoxA1 interactions was calculated by dividing the PD condition values by the non-PD values (Figure 9M, P).

E. 3C Primer Efficiency and Controls

The RP23-329P9 BAC clone, which contains DNA spanning the entire HoxA cluster, was ordered from the Children’s Hospital Oakland Research Institute (CHORI). The clone was Hind III restriction enzyme digested and ligated to generate a HoxA cluster interaction library for testing 3C PCR efficiencies. The primer interaction pairs used for 3C-qPCR were found to all have between 81-96% PCR efficiency. Digestion Efficiency measurements were taken for each 3C preparation by running qPCR on each sample Pre- and Post- Hind III restriction enzyme digestion across each restriction site used for 3C analysis in comparison to a genomic DNA region with no restriction site between its forward and reverse primers (GapDH Primers). Digestion Efficiency was calculated as:

$$Digestion Efficiency =100-\frac{100}{2^((Post Digest across RE CT-Post Digest no RE CT)-(Input across RE CT-Input no RE CT))}$$

Digestion Efficiency measurements were found to all be above 80% with <5% variation between sample conditions for any site. 3C-qPCR results after 40 cycles run on a 2% agarose gel at 90 volts for 50 minutes for ESC and NCC resulted in expected approximately 100-150 bp 3C amplification products for each 3C interaction primer pair measured and the GapDH normalization control.

F. ChIP Sample Preparation

Chromatin Immunoprecipitation (ChIP) samples were collected and prepared using the MAGnify Chromatin Immunoprecipitation System Kit (492024 ThermoFisher, Waltham, MA) with minor alterations. Cells to be harvested for ChIP processing were removed from the incubator and 37% formaldehyde was added to the media for each plate to a final concentration of 1% formaldehyde. The cells were rotated at 40 RPM for 10 minutes at room temperature. 2M glycine was added to each plate to a final concentration of 200 mM to quench the formaldehyde reactions. The plates were rinsed 3x with ice cold DPBS, and then scraped in a volume of 3 ml of DPBS + PI into 15 ml conical tubes. The cells were collected by 1000 RPM centrifugation at 4 $℃$ for 10 minutes. The cells were washed with 500 $\mu$l of DPBS + PI. The cells were collected by 1000 RPM centrifugation and resuspended in 500 $\mu$l MAGnify Lysis Buffer + PI. Sonication was completed on ice using a Branson Sonifier 250 with the Output set at 2 and the Duty set at 30 for 5 intervals of 15 pulses with 1 minute between intervals. Samples were clarified by centrifugation at 16,900 RCF at 4 $℃$ for 5 minutes. The supernatant for each sample was transferred to new sterile 1.5 ml centrifuge tubes. DNA concentrations were approximated using nanodrop to determine how much sample to use for each IP. Each IP was completed using 25 $\mu$g of DNA diluted to 250 $\mu$l with MAGnify Dilution Buffer, 3 $\mu$g of antibody (FGFR1 ab10646 Abcam, Cambridge, MA, CTCF ab70303 Abcam, Cambridge, MA, Rb IgG MAGnify 492024 ThermoFisher, Waltham, MA), and 3 $\mu$l MAGnify Protein A/G Dynabeads and the immunoprecipitations steps were completed according to the manufacturer’s instructions. 10% input samples containing 2.5 $\mu$g DNA were purified separately by RNase A and Proteinase K incubations followed by 65 $℃$ overnight de-crosslinking, phenol chloroform extraction and ethanol precipitation, then dissolved in TE buffer, and the resulting samples were stored at -20 $℃$.

G. ChIP-qPCR Quantification

Three technical replicates were completed on the same plate for each measurement of a primer pair for each biological replicate. Five biological replicates were completed for each sample condition for the nFGFR1 IPs and three biological replicates for the CTCF IPs. The same forward and reverse primer sets were used for both the nFGFR1 and CTCF ChIP-qPCRs. IP sample qPCRs were run on the same plates as their Rb IgG and 10% Input corresponding controls (Figure 9J-K). The measurements that were used for experimental results for each primer pair were calculated as:

$$Percent Input = 100*2^(10\% Input CT - IP sample CT)$$

The fold change effect by PD on ChIP nFGFR1 or CTCF binding was calculated by dividing the PD condition values by the non-PD values (Figure 9N-O, Q-R).

H. mRNA Preparation

Samples for mRNA were collected using the RNeasy Mini Kit (74104 Qiagen, Germantown, MD). Cells were lysed on the plate and collected using cell scrapers. Prior to adding EtOH to the homogenized cell lysate, a 20 $\mu$l aliquot of each sample was taken and treated with RNase A and then phenol chloroform purified to be used later for normalizing each purified RNA sample to their DNA concentration during the reverse transcriptase step of cDNA synthesis. The remainder of the RNeasy preparation was completed using the product recommendations with the purified RNA eluted into 100 $\mu$l of RNase-free dH20. RNA samples were transcribed into cDNA using the iScript one step enzymatic reaction system (1708891 Bio-Rad, Hercules, CA). For cDNA generation, the amount of RNA sample to add to each reaction volume was calculated based on the DNA concentration measurement from each purified DNA aliquot by nanodrop. The iScript cDNA generation reaction PCR protocol was completed following the manufacturer’s instructions and the resulting cDNA samples were stored at -20 $℃$.

I. RT-qPCR Quantification

Three technical replicates were completed on the same plate for each measurement of a primer pair for each biological replicate. Three biological replicates were completed for each sample condition. Prior to cDNA generation, all RNA samples were normalized to the same concentration based on nanodrop concentration measurements of aliquots of their purified DNA taken before the RNA extractions (Figure 9L). To normalize the mRNA qPCR results to values having a baseline of approximately 1 and to show the relative changes between the experimental results, for each primer pair Relative Expression values were calculated as:

$$Relative Expression = [100*2^(-1 *cDNA CT)] * 10E6$$

The fold change effect by PD on mRNA was calculated by dividing the PD condition values by the non-PD values. For these ESC and NCC were compared in Figure 9S. ESC to NCC fold change mRNA in the presence or absence of PD was compared in Figure 9T.

J. Primer Design

3C-qPCR primers were designed flanking the next Hind III restriction site after each of the nFGFR1 ChIP-seq identified binding sites throughout the HoxA cluster [2]. ChIP-qPCR primers were designed to measure binding at each of the HoxA ChIP-seq narrow peak identified binding sites. RT-qPCR primers were designed to each gene transcript (HoxA1-A13) throughout the HoxA gene cluster. Primers were designed for all the experimental procedures using Primer3 online software. The settings were kept the same for the design of all primers whenever possible with the stringency of settings reduced when primer generation was not possible for a location. Product size range was set to 100 – 150 bp. The optimum settings for Primer size was 23, Primer Tm was 60, Primer GC% was 40, and CG Clamp was 1, with all other settings set at their default values. The primers used throughout the experiments are listed in Table S1 Sheet 3.

K. qPCR Routine

Samples were run on a Bio-Rad CFX96 Touch Real-Time PCR Detection System. All qPCR samples were prepared as 20 $\mu$l volumes using 100 nM final primer concentrations (2 $\mu$l volume), 10 $\mu$l 2x SYBR Green Master Mix (1725272 Bio-Rad, Hercules, CA), and 8 $\mu$l of diluted DNA sample. A standard 40 cycle protocol was used for qPCRs for all the experiments. The annealing/extension step was set to 63.0 $℃$ to increase specificity for 3C-qPCR experiments and then was kept the same for qPCRs for the other sample preparation conditions.

1	95.0 $℃$	8:00

2	95.0 $℃$	0:15

3	63.0 $℃$	1:00

Read Plate

4	Go to Step 2 – 39 more times

5	Melt Curve 65.0 to 95.0 $℃$, increment 0.5 $℃$ for 0:05

Read Plate at each increment

End

L. Immunocytochemistry and Microscopy

Double immunostaining was performed according to our established protocol [23, 3] using mouse αFGFR1 (Novus Biologicals, Centennial, CO, 1:200) and rabbit αCTCF (ab84372, 1:500, Abcam, Cambridge, MA). As the secondary antibodies we used anti-mouse Alexa 568 and anti-rabbit Alexa 488. Specificity of immunostaining was ascertained with control reactions in which the primary antibody was omitted or replaced with pre-immune sera, or by neutralizing the antibody with cognate peptide. Staining was observed using a Nikon Diaphot microscope or Bio-Rad MRC 1024 confocal microscope (BioRad Laboratories, Hercules, CA, USA). The possibility of bleed-through in double fluorescent cells was excluded by acquiring images in sequential mode.

M. Statistical Analysis

Statistical approaches were used throughout this study to analyze bins versus other bins in histograms (Z-Score Statistics), ESC histograms versus NCC histograms at the same bins (2-Way ANOVA Tukey), and interaction looping differences between ESC and NCC (Paired t-test). Z-Score Statistics: Two-Tailed Probability from the Central Area was used in assays to identify genomic locations (bins) within the ranges specified (e.g. +/- 1 mb spans) in which the attribute score being analyzed falls outside the normal distribution of the scores for the entire range. The calculation used to determine this was:

$$Z-Score=\frac{(Bin Score-Mean Bin Score)}{Standard Deviation Bin Score}$$

For each separate bin in the entire range of bins, a Z-score greater than 1.96 or less than -1.96 was calculated as having a significance level of p<0.05, with the other higher levels of significance calculated in the same manner. Paired t-tests were used to compare binned interaction scores between ESC and NCC conditions using the t-test function available in the R-stats library, and where specified, the values were corrected using the Bonferroni method for multiple comparisons to adjust p values based on the number of tests performed. Two-Way ANOVA Tukey Method was completed to compare ESC and NCC values for RNA, TAD coverage, and interaction directionality analyses. Each test was completed on 5 – 1 kb values of ESC versus 5 – 1 kb values for NCC using the ANOVA and Tukey Method functions available in R-stats. Two-Way ANOVA with Fisher’s LSD Test was used to determine significance for the qPCR results for the HoxA cluster ESC versus NCC and PD inhibition analyses using the tools available in GraphPad Prism software.

S1 Figure. Chromatin Interactions occur across the genome in both ESC and NCC with controls. (A) Full genome inter- and intra-chromosomal contact map (top) and randomized anchors contact map (bottom). (B) Chromosome 6 intra-chromosomal contact map (left), randomized anchors contact map (middle), and predicted contact map based on read density (right). (C) 49.5-54.0 mb chromosome 6 contact map (right), randomized anchors contact map (middle), and predicted contact map based on read density (right). (D) Chromosome 6 and chromosome 11 data overviews of RNA-seq and nFGFR1 binding and interactions in ESCs and NCCs.

S2 Figure. TAD identification and analyses. (A) Contact maps of HiC libraries at the HoxA locus (arrow) prior to merging. (B) TADs Identified using HiCtool in ESC and NCC are shown stacked in rows as a cumulative plot. TADs in ESC and NCC are similar in number and size but the locations of TADs change. (C) Same size TAD analysis - 200 TADs of size 480kb (yellow box) are sorted by interaction anchor strength for comparison to other attributes. (D) All TADs analysis - TADs are split in half (yellow line) and aligned to their left and right borders. For the TADs >2 mb, the middle portion of the TAD is removed leaving behind only + /- 1 mb of left and right TAD regions. (E) Genome-wide and chromosome 6 overviews with ESC interactions and an interchromosomal spectral color scheme. (F) 1 kb binned genome data structure - for each attribute which contain a score and a location (interactions, nFGFR1 binding, RNA expression) the scores were summed at the genome location bins. For attributes which are only a location (Promoters, Exons, CpG Islands, and other coding and regulatory features) their bp spans were summed for each bin within which they occur. (G) Regulated TADs analysis – regulated TADs were identified based on the midpoints between anchors of high confidence interaction peaks (q<0.001) containing regulated genes in their anchors.

S3 Figure. Chromatin intra- and inter-chromosomal interactions change between ESC and NCC. (A) Full genome and (B) enhanced view locations of stronger ESC and NCC interactions in 1 mb interaction bins thresholded by paired t-test p<0.05. Raw interactions for ESC and NCC intra- (empirical distribution p<0.05) and NCC inter- (empirical distribution p<0.1) interactions are shown in the inner tracks to indicate regions of strong interactions for comparison to the relative differences. (C) 5000 p<0.05 thresholded interactions ranked by p value for ESC or NCC intra- or inter-chromosomal stronger interactions. Line thickness shows interaction strength changes between ESC and NCC.

S4 Figure. nFGFR1 and Combinations of attributes predict interaction strength in the genome. Predicting interaction score was completed using two output classes based on whether the interaction score value was below or above the average interaction score in 150/220 kb windows.

S5 Figure. Interaction strength correlates with gene coding, regulatory features, and nFGFR1 in NCC TADs. 200 TADs of 480 kb stacked as rows in descending order by interaction strength within each TAD. Heatmaps show the feature span (gene coding and regulatory features) or strength (interaction strength, RNA FPKM, nFGFR1 binding) throughout each TAD and a mean plot of the heatmap rows in 5 TAD bins. Pearson’s R values were calculated between interaction strength and each of the corresponding attributes in 5 TAD bins. Right panels to each heatmap show average interaction strength sorted feature enrichments across same sized TADs. Z-Score statistics indicate bins which are outside the mean of all bins p<0.05 ▂ ; p<0.01 ▃ ; p<0.005 ▅ .

S6 Figure. Additional TAD analyses 1. (A-H) All TADs in NCC split in half and aligned by left and right TAD borders (NCBI bp notation directionality). Heatmaps show interaction strength, RNA FPKM, nFGFR1 binding, and gene coding and regulatory features enrichments in each TAD. (I-N) All TADs in ESC and NCC for FGFR1 and CTCF HiChIP anchor strength enrichment. Top and right panels to each heat map show average location (column means – 5 kb bins) and size (row means – 5 TAD bins) dependent enrichment of the features across all TADs. Z-Score statistics indicate bins which are outside the mean of all bins p<0.05 ▂ ; p<0.01 ▃ ; p<0.005 ▅ .

**S7 Figure.** **Additional TAD analyses 2.** (A) DiffTAD analysis with HiC Strength thresholded by hichipper q<0.001. (B) DiffTAD analysis with 400 locations analyzed. (C) Neuronal Gene Ontology category enrichment in down- and upregulated TADs. Z-Score statistics indicate bins which are outside the mean of all bins p<0.05 ▂ ; p<0.01 ▃ ; p<0.005 ▅ .

**Inside TADs**

S8 Figure. NCC- 5’ TAD Border Overrepresentation of Motifs. Motif overrepresentation measured + - 195 kb surrounding borders of NCC- gene containing TADs in 10 kb bins. Motif Relative Occurrence is calculated by the mean count of each motif from 50 iterations of R-GADEM motif search results on 10 runs of random sampling of 1000 locations of 100 bp length within each 10 kb bin, divided by the average motif count from all the bins together. The motifs are numbered in descending order by the average number of times the motif occurred throughout the + - 195 kb region. Motifs CTCF, GABPA, GATA2, HNF1A, NFYA, Nkx3.2, and others, were found overrepresented in the NCC- 5’ TAD domain borders regions. The x-axis indicates the distance + - from aligned TAD borders. The y-axis indicates the overrepresentation of protein binding motifs at each location vs all other locations in the + - 195 kb region as a cumulative plot (values greater than or less than 1 indicating over- or under- representation of the motif for that location bin). Z-Score statistics indicate bins which are outside the mean of all bins, p<0.05: ○; p<0.01: 〇; p<0.005: ◯; Bonferroni adjusted p<0.05: +.

**Inside TADs**

S9 Figure. NCC- 3’ TAD Border Overrepresentation of Motifs. Motif overrepresentation measured + - 195 kb surrounding borders of NCC- gene containing TADs in 10 kb bins. Motif Relative Occurrence is calculated by the mean count of each motif from 50 iterations of R-GADEM motif search results on 10 runs of random sampling of 1000 locations of 100 bp length within each 10 kb bin, divided by the average motif count from all the bins together. The motifs are numbered in descending order by the average number of times the motif occurred throughout the + - 195 kb region. Motifs Arnt, CTCF, MAX, MYC..MAX, Pou5f1, Prrx2, and others, were found overrepresented in the NCC- 3’ TAD domain borders regions. The x-axis indicates the distance + - from aligned TAD borders. The y-axis indicates the overrepresentation of protein binding motifs at each location vs all other locations in the + - 195 kb region as a cumulative plot (values greater than or less than 1 indicating over- or under- representation of the motif for that location bin). Z-Score statistics indicate bins which are outside the mean of all bins, p<0.05: ○; p<0.01: 〇; p<0.005: ◯; Bonferroni adjusted p<0.05: +.

**Inside TADs**

S10 Figure. NCC+ 5’ TAD Border Overrepresentation of Motifs. Motif overrepresentation measured + - 195 kb surrounding borders of NCC+ gene containing TADs in 10 kb bins. Motif Relative Occurrence is calculated by the mean count of each motif from 50 iterations of R-GADEM motif search results on 10 runs of random sampling of 1000 locations of 100 bp length within each 10 kb bin, divided by the average motif count from all the bins together. The motifs are numbered in descending order by the average number of times the motif occurred throughout the + - 195 kb region. Motifs CREB1, CTCF, En1, Evi1, HOXA5, Mafb, and others, were found overrepresented in the NCC+ 5’ TAD domain borders regions. The x-axis indicates the distance + - from aligned TAD borders. The y-axis indicates the overrepresentation of protein binding motifs at each location vs all other locations in the + - 195 kb region as a cumulative plot (values greater than or less than 1 indicating over- or under- representation of the motif for that location bin). Z-Score statistics indicate bins which are outside the mean of all bins, p<0.05: ○; p<0.01: 〇; p<0.005: ◯; Bonferroni adjusted p<0.05: +.

**Inside TADs**

S11 Figure. NCC+ 3’ TAD Border Overrepresentation of Motifs. Motif overrepresentation measured + - 195 kb surrounding borders of NCC+ gene containing TADs in 10 kb bins. Motif Relative Occurrence is calculated by the mean count of each motif from 50 iterations of R-GADEM motif search results on 10 runs of random sampling of 1000 locations of 100 bp length within each 10 kb bin, divided by the average motif count from all the bins together. The motifs are numbered in descending order by the average number of times the motif occurred throughout the + - 195 kb region. Motifs CTCF, E2F1, ELK4, Foxa2, FOXO3, Myc, and others, were found overrepresented in the NCC+ 3’ TAD domain borders regions. The x-axis indicates the distance + - from aligned TAD borders. The y-axis indicates the overrepresentation of protein binding motifs at each location vs all other locations in the + - 195 kb region as a cumulative plot (values greater than or less than 1 indicating over- or under- representation of the motif for that location bin). Z-Score statistics indicate bins which are outside the mean of all bins, p<0.05: ○; p<0.01: 〇; p<0.005: ◯; Bonferroni adjusted p<0.05: +.

S12 Figure. TAD Border Overrepresentation of Motifs Individual Facet Graphs. Motif overrepresentation shown as individual plots. Motif overrepresentation measured + - 195 kb surrounding borders of NCC gene containing TADs in 10 kb bins. Motif Relative Occurrence is calculated by the mean count of each motif from 50 iterations of R-GADEM motif search results on 10 runs of random sampling of 1000 locations of 100 bp length within each 10 kb bin, divided by the average motif count from all the bins together. The x-axis indicates the distance + - from aligned TAD borders. The y-axis indicates the overrepresentation of protein binding motifs at each location vs all other locations in the + - 195 kb region as a cumulative plot (values greater than or less than 1 indicating over- or under- representation of the motif for that location bin). Z-Score statistics indicate bins which are outside the mean of all bins, p<0.05: ●; p<0.01: ⚫; p<0.005: ⚫; Bonferroni adjusted p<0.05: +.

S13 Figure. Structurally and Developmentally Associated Motifs are Targeted by nFGFR1 in NCC Regulated TAD Borders. nFGFR1 targeted binding motifs were identified + - 100 kb at 5’ and 3’ borders of NCC regulated TADs. nFGFR1 Binding Preference indicates the relative number of times each motif occurred in comparison to the other identified motifs, and is calculated by the mean count of each motif as a part of a 1.0 whole of all the nFGFR1 bound motif counts added together from 10 iterations of R-GADEM motif search results on 10 runs of random ordering of the nFGFR1 narrow peak binding sites within + -100 kb of each border (~ 200-400 peaks per border of size approx. 150 bp). The motifs are ordered alphabetically with the colored square above each motif indicating the nFGFR1 Binding Preference. (A-D) NCC Downregulated TADs nFGFR1 motif targeting in 5’ and 3’ borders in ESC and NCC. (E-H) NCC Upregulated TADs nFGFR1 motif targeting in 5’ and 3’ borders in ESC and NCC.

S14 Figure. HoxA and HoxC clusters connect interchromosomally in ESC and NCC, and HoxA and HoxD in ESC. The interchromosomal interactions reorganize during NCC differentiation. (A-H) Interchromosomal contact maps showing whole chromosome interactions between Chr6 and Chr15 with the HoxA-HoxC interaction block circled (A-B) and Chr6 and Chr2 with the HoxA-HoxD interaction block circled (E-F) and enhanced views of the circled region with (+) indicating the intersections between the Hox clusters (C-D and G-H).

S15 Figure. Hox clusters reorganize during NCC differentiation in parallel with gene expression and nFGFR1 binding changes. (A-D) Intrachromosomal contact maps of the HoxA-D clusters showing ESC and NCC interactions, q<0.05 Log2 Fold Change RNA FPKM, and nFGFR1 ChIP-seq narrow peak locations. (E-F) HoxC and HoxD Differential looping. Paired T-Test Bonferroni adjusted p<10^-5^ ▂ ; p<10^-15^ ▃ ; p<10^-25^ ▅ . (G-H) Differential interaction anchor strength. (I-J) nFGFR1 binding. (K-L) Differential gene expression.

S16 Figure. Hox clusters reorganize during NCC differentiation with contributions of nFGFR1 and CTCF. (A-D) HoxA - HoxD Differential looping (top rows) in comparison to Diffferential nFGFR1 looping (middle rows) and Differential CTCF looping (bottom rows). Paired t-test Bonferroni adjusted p<10^-5^ ▂ ; p<10^-15^ ▃ ; p<10^-25^ ▅ .
